# Esketamine vs Midazolam in Boosting the Efficacy of Oral Antidepressants for Major Depressive Disorder: A Pilot Randomized Clinical Trial

**DOI:** 10.1001/jamanetworkopen.2023.28817

**Published:** 2023-08-14

**Authors:** Chunfeng Xiao, Jia Zhou, Anning Li, Ling Zhang, Xuequan Zhu, Jingjing Zhou, Yongdong Hu, Yunying Zheng, Jing Liu, Qiying Deng, Haibo Wang, Gang Wang

**Affiliations:** 1 Beijing Key Laboratory of Mental Disorders, National Clinical Research Center for Mental Disorders & National Center for Mental Disorders, Beijing Anding Hospital, Capital Medical University, Beijing, China; 2 Advanced Innovation Center for Human Brain Protection, Capital Medical University, Beijing, China; 3 Unit of Psychological Medicine, Beijing Chao-Yang Hospital, Capital Medical University, Beijing, China; 4 Peking University Clinical Research Institute, Peking University First Hospital, Beijing, China; 5 Key Laboratory of Epidemiology of Major Diseases, Peking University, Ministry of Education

## Abstract

**Importance:**

Loss of a previously effective response while still using adequate antidepressant treatment occurs in a relatively high proportion of patients with major depressive disorder (MDD); therefore, there is a need to develop novel effective treatment strategies.

**Objective:**

To assess the efficacy and safety of a single subanesthetic dose of esketamine in boosting the efficacy of oral antidepressants for treating fluctuating antidepressant response in MDD.

**Design, Setting, and Participants:**

This single-center, double-blind, midazolam-controlled pilot randomized clinical trial was conducted at Beijing Anding Hospital, Capital Medical University in China. The study enrolled participants aged 18 years and older with fluctuating antidepressant response, defined as patients with MDD experiencing fluctuating symptoms after symptom relief and stabilization. Patient recruitment was conducted from August 2021 to January 2022, and participants were followed-up for 6 weeks. Data were analyzed as intention-to-treat from July to September 2022.

**Interventions:**

All participants in the esketamine-treated group received intravenous esketamine at 0.2 mg/kg in 40 minutes. Participants in the midazolam control group received intravenous midazolam at 0.045 mg/kg in 40 minutes.

**Main Outcomes and Measures:**

The primary outcome was the response rate at 2 weeks, defined as a 50% reduction in Montgomery-Åsberg Depression Rating Scale (MADRS). Secondary outcomes included response rate at 6 weeks, remission rates at 2 and 6 weeks, and change in MADRS and Clinical Global Impression–Severity score from baseline to 6 weeks; remission was defined by a MADRS score of 10 or lower.

**Results:**

A total of 30 patients (median [IQR] age, 28.0 [24.0-40.0] years; 17 [56.7%] female) were randomized, including 15 patients randomized to midazolam and 15 patients randomized to esketamine; 29 patients completed the study. Response rates at 2 weeks were significantly higher in the esketamine-treated group than in the midazolam control group (10 patients [66.7%] vs 1 patient [6.7%]; *P* < .001). Participants treated with esketamine experienced significantly greater reduction in MADRS score from baseline to 2 weeks compared with those treated with midazolam (mean [SD] reduction, 15.7 [1.5] vs 3.1 [1.3]; *P* < .001). No serious adverse events were observed in this trial, and no psychotogenic effects and clinically significant manic symptoms were reported.

**Conclusions and Relevance:**

This pilot randomized clinical trial found that a single subanesthetic dose of esketamine could boost the efficacy of oral antidepressants in treating fluctuating antidepressant response, with a good safety profile.

**Trial Registration:**

Chinese Clinical Trial Registry Identifier: ChiCTR2100050335

## Key Points

**Question:**

Can esketamine boost the efficacy of oral antidepressants in patients with major depressive disorder with fluctuating symptoms (ie, reemergence of initial symptoms or episode or onset of a new episode) occurring during adequate antidepressant treatment?

**Findings:**

In this randomized clinical trial of 30 patients with fluctuating antidepressant response, more patients who received a single subanesthetic dose of esketamine experienced a reduction of depression severity by at least 50% compared with patients who received midazolam, The difference remained statistically significant at the final follow-up at 6 weeks.

**Meaning:**

These findings suggest that esketamine could be used as a potentially effective and safe treatment option for fluctuating antidepressant response.

## Teaser

This randomized clinical trial assesses the efficacy and safety of a single subanesthetic dose of esketamine compared with midazolam control in boosting the efficacy of oral antidepressants for treating fluctuating antidepressant response in major depressive disorder.

## Introduction

Depression is a common chronic and disabling condition that affects an estimated 300 million people worldwide and is associated with personal, societal, and economic burdens.^1,2,3^ Depression has high rates of recurrence and disability, with approximately half of patients experiencing a relapse or a recurrence.^4,5,6^

Pharmacotherapy is currently the leading strategy in the treatment of major depressive disorder (MDD).^7,8^ Treatment guidelines recommend that patients with MDD continue antidepressant therapy for 4 to 9 months after successful acute phase treatment to prevent relapse and recurrence and 2 years or more of maintenance treatment at a full therapeutic dose for patients with an increased risk of MDD recurrence.^9,10^ However, rates of relapse and recurrence remain high even among adherent patients with MDD.^11,12,13^

Unlike relapse and recurrence, which occur during the continuation or maintenance phase, loss of a previously effective response while still receiving adequate antidepressant treatment (ADT) occurs in a relatively high proportion of patients with MDD, with rates ranging from 9% to 57%.^14^ This concerning phenomenon is known as *ADT tachyphylaxis* or *antidepressant tolerance*.^15^ Tachyphylaxis is a pharmacological term defined as the rapid appearance of a progressive decrease in response to a given dose after administering an active substance.^16^ This metric is unconducive to our examination of this phenomenon from a clinical perspective in practice, especially when this phenomenon is not well understood. Instead, we used the term *fluctuating antidepressant response in MDD* (FAD) to refer to the occurrence of fluctuating symptoms, including re-emergence of initial symptoms or episodes or the onset of a new episode during adequate ADT in patients with MDD.

The causes of FAD remain unclear, and there is a lack of effective treatments.^14^ Available options are limited to increasing the current antidepressant dose, drug holidays, decreasing current antidepressant dose, changing antidepressant drugs, augmenting treatment strategies, and combination treatment strategies. These strategies might be successful after several attempts, but they increase the risk of unanticipated adverse effects in the meantime.^15,17^ FAD may contribute to treatment-resistant depression (TRD) development.^18,19^ Therefore, finding novel effective treatment strategies is essential to prevent TRD and adverse outcomes in patients with MDD.

Ketamine, a racemic mixture of 2 enantiomers (esketamine and R-ketamine), is a noncompetitive N-methyl-D-aspartate (NMDA) receptor antagonist that has been used in clinical practice as a standard anesthetic for nearly half a century.^20^ It has been demonstrated that 40-minute administration of 0.5 mg/kg ketamine or 0.2 mg/kg esketamine produced a more pronounced antidepressant effect than other subanesthetic intravenous doses.^21,22,23,24^ Ketamine has an affinity for multiple receptors, including the NMDA receptor, opioid receptor, and alpha-amino-3-hydroxy-5-methyl-4-isoxazole propionic acid receptor, so it can initiate cascading effects acting in concert.^22,25,26,27^ Since ketamine can act on the same receptors as current antidepressants, combining ketamine with existing antidepressants may be more beneficial for patients with MDD.^26^ A study by Hu et al^27^ reported that a single subanesthetic dose of ketamine could shorten antidepressant onset. Moreover, a recent clinical study found that ketamine may interact with existing antidepressants. Another study by Popova et al^28^ found that switching patients with TRD from an ineffective antidepressant to a flexibly dosed esketamine nasal spray plus a newly initiated antidepressant was more effective than the newly initiated antidepressant plus placebo. Thus, ketamine might potentially boost the efficacy of oral antidepressants. Therefore, we conducted a double-blind, midazolam-controlled randomized clinical trial to explore the efficacy and safety of a single dose subanesthetic esketamine plus ongoing adequate ADT among patients with FAD.

## Methods

### Trial Design

This single-center, double-blind, midazolam-controlled randomized clinical trial was conducted at Beijing Anding Hospital, Capital Medical University, Beijing, China, and approved by a local independent ethics committee. All participants provided their written informed consent. This study followed the Consolidated Standards of Reporting Trials (CONSORT) reporting guideline. The trial protocol and statistical analysis plan are provided in Supplement 1.

### Patients

Key inclusion criteria were age 18 to 60 years and diagnosed MDD according to the *Diagnostic and Statistical Manual of Mental Disorders* (Fifth Edition). Symptoms were measured with 2 questions on a visual analogue scale (VAS; range, 0-10; lower score indicates worse symptoms): question 1, “How did you feel about your state during the period when your condition was stable, under the treatment of your current medication?” and question 2, “How do you feel about your state during the recent period when you are not feeling well, under the treatment of your current medication?” Patients were eligible for inclusion if they were experiencing symptom fluctuation (VAS question 2 score, ≤5) after symptom relief and stabilization (VAS question 1 score, ≥8). For inclusion, the patient’s most recent depressive episode had to have been stabilized by a single antidepressant without any adjustments in the type and dosage, and the duration of drug interruption during this period had to be less than 7 days. Finally, patients must have had a score of at least 14 on the 17-item Hamilton Rating Scale for Depression (range, 0-52, with higher score indicating more severe depression symptoms). The key exclusion criteria were (1) a history of TRD, schizophrenia, bipolar disorder, obsessive-compulsive disorder, drug or alcohol dependence, and ketamine or its enantiomers exposure, or current MDD episode with psychotic features; (2) contraindications to esketamine or midazolam; (3) clinically significant anomalies in laboratory test results at screening and any concern of certain conditions with trial risks. Detailed eligibility criteria are described in Supplement 1.

### Randomization and Blinding

Eligible patients were randomly assigned in a 1:1 ratio to receive either intravenous esketamine or intravenous midazolam for 40 minutes using a computer-generated block randomization scheme (block size, 4). An independent assistant who was not involved in the study was responsible for packing and labeling the prescribed medication. Study investigators, staff, participants, and all outcome assessors were blinded to the treatment assignment.

### Interventions

The infusion process was performed at Beijing Anding Hospital, Capital Medical University. All participants were admitted to the research ward the day before infusion and started 6 hours preoperative fasting and water deprivation. On the infusion day, 0.2 mg/kg esketamine or 0.045 mg/kg midazolam was administered intravenously according to randomization group. Both medications were dissolved in 50 mL of normal saline and administered for 40 minutes using a syringe pump. Safety checks were performed before, during, and after infusion. Detailed information on the safety check procedure is presented in the trial protocol in Supplement 1. Participants were permitted to discharge after completing the 24 hours follow-up visit after infusion. All participants in both groups maintained their original drug regimen throughout the study.

### Efficacy Assessments

Efficacy was assessed by the Montgomery-Åsberg Depression Rating Scale (MADRS; range, 0-60; higher scores indicating more severe depressive symptoms) and Clinical Global Impression–Severity (CGI-S; range, 1-7; higher score indicates greater severity of illness) at 40 minutes; 2, 4, and 24 hours; and 1, 2, 4, and 6 weeks after infusion. Recalling time of MADRS general version is 1 week. In this regard, the structured interview guide for MADRS was used at 4 and 24 hours with its corresponding version (only these versions were available before this study), since such guides have previously been shown to increase the reliability of given scales and to ensure the accuracy of assessment a short time after infusion.^29^

### Safety Assessments

General side effects were measured by the Udvalg for Kliniske Undersogelser side effect rating scale (range, 0-144; higher score indicates more severe side effects) and the Frequency, Intensity, and Burden of Side Effects Rating Scale (range, 0-18; higher score indicates more severe side effects). Psychotogenic effects were measured with the 4-item positive symptom subscale of Brief Psychiatric Rating Scale consisting of suspiciousness, hallucinations, unusual thought content, and conceptual disorganization (range, 4-28; higher score indicates more severe psychotic symptoms). Dissociative effects were measured with the Clinician-Administered Dissociative States Scale (range, 0-92; higher score indicates more severe dissociative symptoms), and manic symptoms were assessed using the Young Mania Rating Scale (range, 0-60; higher score indicates more severe manic symptoms). The Columbia-Suicide Severity Rating Scale (range, 0-42; higher score indicating greater suicidal risk) was also rated throughout the trial for safety purposes.

### Outcomes

The primary outcome was the response rate at 2 weeks, with response defined as a 50% reduction in the MADRS. Secondary outcomes included the response rate at 6 weeks, remission rates at 2 and 6 weeks, and a change in MADRS and CGI-S score from baseline to 6 weeks, with remission defined by a MADRS score of 10 or lower. Safety outcomes included general side effects, psychotogenic effects, dissociative effects, manic symptoms, and suicide risk.

### Sample Size Calculation

Since this study was a pilot study, no formal sample size calculation was performed. We planned to recruit 30 participants (15 participants in each group). Effect size data from this pilot study will be used to estimate the sample size of a future randomized clinical trial.

### Statistical Analysis

Analyses were performed as intention-to-treat, including all randomized participants who completed the assessments at baseline and received study medication (full analysis set [FAS]). Missing data for MADRS scores were imputed using the last observation carried forward. Continuous variables are presented as mean (SD) or median (IQR). Categorical variables are presented as numbers and percentages. We used χ^2^ tests, Fisher exact test, independent-sample *t* test, or Wilcoxon rank-sum test, as appropriate, to compare the differences in baseline variables between the intervention and control groups. Treatment effects (ie, response and remission rates) were evaluated using the χ^2^ test. Sensitivity analysis was also performed for the primary outcome using the logistic regression model with baseline MADRS score and the number of MDD episodes as covariates. Secondary outcomes, the reduction in MADRS score and CGI-S from baseline over time, were analyzed using independent-sample *t* tests or Wilcoxon rank-sum test. Sensitivity analysis was also performed using an analysis of covariance model with baseline MADRS score and the number of MDD episodes as covariates. Moreover, to compare the difference between groups over time, a 2-way repeated measures analysis of variance was performed accounting for group, time, and the interaction term between group and time. All tests were 2-sided, and *P* < .05 was considered statistically significant. All the statistical analyses were performed with SAS statistical software, version 9.4 (SAS Institute). Data were analyzed from July to September 2022.

## Results

### Participants

**Figure 1.**
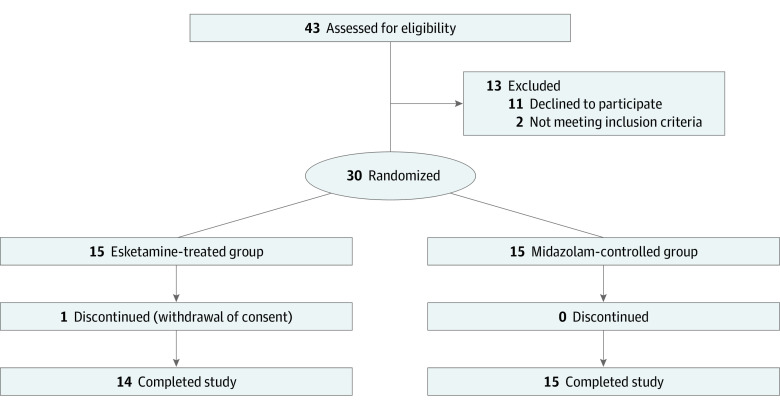
Participant Recruitment Flowchart One patient in the esketamine-treated group dropped out at the end of 1 week. They received 0.2 mg/kg esketamine infusion and left the city due to job changes.

From August 2021 to January 2022, 43 participants were evaluated for eligibility, and 30 participants (median [IQR] age, 28.0 [24.0-40.0] years; 17 [56.7%] female) with FAD were enrolled (Figure 1). All the enrolled participants were included in the FAS for final analysis, and 29 participants (96.7%) completed the trial. There was 1 participant in the esketamine-treated group who dropped out at the end of 1 week. They received 0.2 mg/kg esketamine infusion and left the city due to a job change.

The 2 groups were generally balanced in terms of baseline demographic and clinical characteristics, except for the proportion of participants with MDD of the first episode, mean number of MDD episodes, and mean MADRS score (Table 1). The median (IQR) duration of ongoing ADT with their current antidepressant was 24.9 (14.9-51.0) weeks, 8.6 (4.0-32.9) weeks stable after symptom relief, and 5.8 (3.1-10.3) weeks with fluctuating symptoms. Among all participants, 15 (50.0%) were experiencing their first episode of MDD, 5 (16.7%) had a family history of psychiatric illness, and 19 (63.3%) were receiving selective serotonin receptor inhibitors, followed by 9 (30.0%) receiving serotonin and norepinephrine reuptake inhibitors, and 2 (6.7%) receiving other types of antidepressants.

**Table 1.** Participant Demographic and Clinical Characteristics at Baseline

Characteristic	Participants, No. (%)	
	Midazolam-controlled group (n = 15)	Esketamine-treated group (n = 15)
Sex		
Female	8 (53.3)	9 (60.0)
Male	7 (46.7)	6 (40.0)
Ethnicity		
Han	15 (100)	13 (86.7)
Other^a^	0	2 (13.3)
Education level, y		
>12	12 (80.0)	13 (86.7)
≤12	3 (20.0)	2 (13.3)
Marital status		
Married	5 (33.3)	6 (40.0)
Unmarried	10 (66.7)	9 (60.0)
Alcohol history		
No	11 (73.3)	11 (73.3)
Yes	4 (26.7)	4 (26.7)
Smoking history		
No	10 (66.7)	11 (73.3)
Yes	5 (33.3)	4 (26.7)
Family history of psychiatric illness		
No	12 (80.0)	13 (86.7)
Yes	3 (20.0)	2 (13.3)
First-episode MDD		
No	11 (73.3)	4 (26.7)
Yes	4 (26.7)	11 (73.3)
Antidepressant use		
Bupropion	1 (6.7)	0 (0.0)
Escitalopram oxalate	4 (26.7)	4 (26.7)
Duloxetine	1 (6.7)	2 (13.3)
Fluvoxamine	1 (6.7)	2 (13.3)
Fluoxetine	1 (6.7)	1 (6.7)
Mirtazapine	0	1 (6.7)
Sertraline	3 (20.0)	3 (20.0)
Venlafaxine	4 (26.7)	2 (13.3)
Class of antidepressant		
SSRIs	9 (60.0)	10 (66.7)
SNRIs	5 (33.3)	4 (26.7)
Other^b^	1 (6.7)	1 (6.7)
Age, median (IQR), y	26.0 (24.0-37.0)	30.0 (25.0-40.0)
BMI, mean (SE)	24.1 (5.9)	23.7 (3.7)
Age at onset of MDD, mean (SE), y	23.9 (8.1)	28.5 (11.7)
Total course of MDD, median (IQR), y	5.0 (1.8-12.6)	1.9 (0.6-6.7)
MDD episodes, mean (SE), No.	3.0 (1.0-6.0)	1.0 (1.0-2.0)
Duration of recent episode, median (IQR), wk	48.0 (29.0-67.0)	61.0 (26.0-103.0)
Duration of ADT for recent episode, median (IQR), wk	30.3 (11.7-52.7)	59.6 (20.9-99.0)
Duration of symptoms with current antidepressant medication, median (IQR), wk		
Ongoing ADT	26.4 (11.7-45.7)	23.1 (14.9-85.4)
Maintaining stability after symptom relief	11.0 (3.3-31.6)	8.0 (4.0-36.0)
Fluctuating symptoms	6.6 (3.1-14.1)	4.7 (2.3-10.0)
Screening HAMD-17 score, mean (SE)^c^	18.9 (4.2)	20.1 (2.6)
Baseline MADRS score, mean (SE)^d^	21.3 (5.0)	26.1 (6.4)
Screening VAS-1 score, median (IQR)^e^	8.0 (8.0-9.0)	8.0 (8.0-8.0)
Screening VAS-2 score, median (IQR)^e^	2.8 (1.2)	2.7 (1.1)

Abbreviations: ADT, antidepressant treatment; BMI, body mass index (calculated as weight in kilograms divided by height in meters squared); HAMD-17, 17-item Hamilton Rating Scale for Depression; MADRS, the Montgomery-Åsberg Depression Rating Scale; MDD, major depressive disorder; SNRIs, serotonin and norepinephrine reuptake inhibitors; SSRIs, selective serotonin reuptake inhibitors; VAS, Visual Analogue Scale score.

^a^ Two patients were included in other ethnicities: 1 identified as Hui ethnicity, and the other identified as Man ethnicity.

^b^ One patient was receiving a norepinephrine–dopamine reuptake inhibitor (bupropion), and the other was receiving a noradrenergic and specific serotonergic antidepressant (mirtazapine).

^c^ Range, 0 to 52; higher total score indicates more severe depression.

^d^ Range, 0 to 60; higher score indicates more severe depressive symptoms.

^e^ Range, 0 to 10. A score of 0 indicates “Seeing my normal state as a reference, my state is horrible during this period”; 10, “Seeing my normal state as a reference, my state is quite good (close to my normal state)” during this period. VAS-1 was “How did you feel about your state during the period when your condition was stable, under the treatment of your current medication?”; VAS-2 was “How do you feel about your state during the recent period when you are not feeling well, under the treatment of your current medication?”

### Primary Outcome

**Figure 2.**
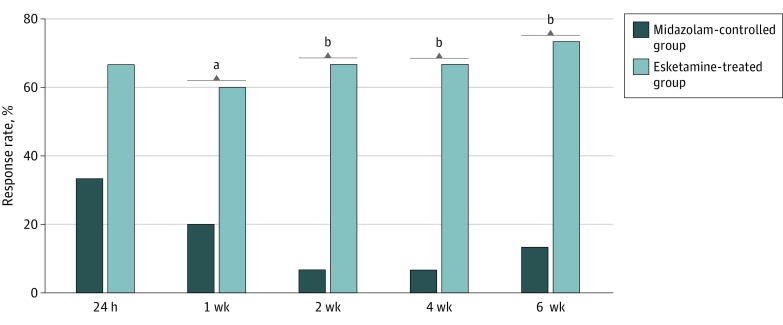
The Response Rates at Different Visits by Intervention and Control Group Response was defined as a 50% reduction in the Montgomery-Åsberg Depression Rating Scale (MADRS; range, 0-60; higher scores indicating more severe depressive symptoms). Missing data for 1 patient’s MADRS score were imputed using the last observation carried forward. ^a^*P* < .05. ^b^*P* < .001.

At 2 weeks, the response rate was significantly higher in the group treated with esketamine than in participants in the midazolam control group (10 participants [66.7%] vs 1 participants [6.7%]; OR, 28.00; 95% CI, 2.82 to 277.96). Significant between-group differences were also observed at other visits, including 1, 4, and 6 weeks (Figure 2). However, there was no significant difference in response rate between the intervention and control groups at 24 hours (10 participants [66.7%] vs 5 participants [33.3%]; *P* = .07). In sensitivity analysis using logistic regression, the difference in response rate between the esketamine-treated and midazolam control groups at 2 weeks remained statistically significant after adjusting for the baseline MADRS score and the number of MDD episodes (OR, 14.17; 95% CI, 1.14 to 175.96; *P* = .04).

### Secondary Outcomes

Participants in the esketamine-treated group had higher remission rates than the midazolam control group at 4 hours (10 participants [66.7%] vs 3 participants [20.0%]; *P* = .01) and 4 weeks (8 participants [53.3%] vs 1 participants [6.7%]; *P* = .005). There was no statistically significant difference between groups at other visits **(**eTable 1 in Supplement 2).

**Figure 3.**
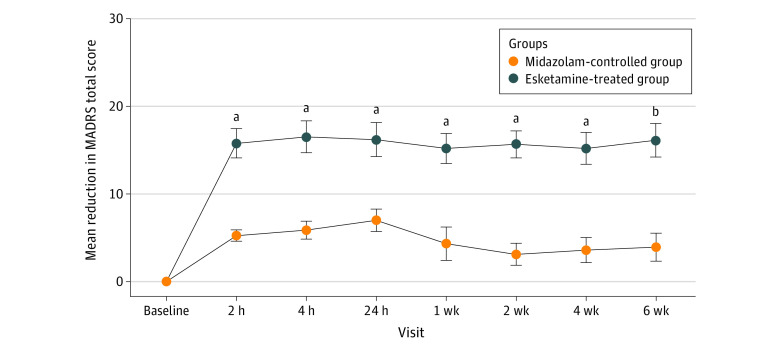
The Mean Reduction of Montgomery-Åsberg Depression Rating Scale (MADRS) Scores From Baseline to 6 Weeks at Each Visit by Intervention and Control Group MADRS (the Montgomery-Åsberg Depression Rating Scale; range: 0-60; higher scores indicating more severe depressive symptoms). Error bars indicate SEs. Missing data of MADRS score for 1 patient were imputed using the last observation carried forward. ^a^*P* < .001. ^b^*P* < .01.

Mean reduction in MADRS score from baseline to 2 weeks among participants treated with esketamine was significantly greater than among those treated with midazolam (mean [SD] reduction, 15.7 [1.5] vs 3.1 [1.3]; *P* < .001). This between-group difference was also observed at all other visits (Figure 3). Additionally, results of analysis of covariance indicated that differences between groups in the reduction in MADRS score from baseline to 6 weeks remained statistically significant after adjusting for the baseline MADRS score and the number of MDD episodes, except for at 24 hours. After adjustment for the covariates, the differences in the least squares mean reduction between the groups were −8.74 (95% CI, −13.11 to- −4.37; *P* < .001) at 2 weeks and −5.55 (95% CI, −10.08 to −1.03; *P* = .02) at 6 weeks (eFigure in Supplement 2). The results of 2-way repeated-measures analysis of variance indicated that the difference between groups over time was statistically significant, referring to interaction between factors (time × group) (*F* = 8.27; *P* < .001). The reduction in CGI-S score from baseline to 6 weeks was significantly greater in the esketamine-treated group than in the midazolam control group at each visit (eTable 2 in Supplement 2).

### Safety

No serious adverse events were observed in this trial, no psychotogenic effects were reported on the Brief Psychiatric Rating Scale, and no clinically significant manic symptoms were reported on the Young Mania Rating Scale. All participants scored 0 points in Clinician-Administered Dissociative States Scale at 2, 4, and 24 hours. At 40 minutes, participants in the esketamine-treated group showed higher level of dissociation compared with the midazolam control group (median [IQR] score, 0 [0-0] vs 6 [3-13]; *P* < .001). For general side effects assessed by Udvalg for Kliniske Undersogelser, which were defined as an increase of at least 1 on each item, 2 participants (13.3%) had increased dream activity in the group treated with esketamine, and 4 participants (26.7%) had tension or inner unrest and 3 participants (20.0%) had increased dream activity in the midazolam control group (Table 2).

**Table 2.** Reported Frequency of Side Effects and Adverse Events Assessed With Udvalg for Kliniske Undersogelser by Intervention and Control Groups

Side effect	Participants, No.	
	Midazolam control group	Esketamine-treated group
Psychiatric		
Asthenia or lassitude	0	1
Sleepiness or sedation	0	1
Tension or inner unrest	4	0
Reduced duration of sleep	0	1
Increased dream activity	3	2
Emotional indifference	1	1
Neurologic: tremors	2	0
Autonomic		
Polyuria or polydipsia	1	0
Palpitations or tachycardia	1	0
Increased sweating	1	0
Other		
Weight loss	0	1
Reduced sexual desire	1	0

## Discussion

This double-blind, pilot randomized clinical trial assessed the efficacy and safety of a subanesthetic dose of esketamine for treating FAD and found that esketamine was more effective than midazolam, with a good safety profile. Efficacy duration of a single subanesthetic dose of esketamine for treating MDD, including TRD, is approximately 10 to 14 days, with limited studies suggesting longer-lasting effects.^27^ The most recent evidence indicates that the efficacy of esketamine as an add-on therapy in combination with oral antidepressants does not extend beyond 4 weeks. In a double-blind, active-controlled randomized clinical trial by Chen et al,^30^ the group receiving esketamine exhibited a clinically meaningful treatment effect at 24 hours compared with the placebo group, but the effect had diminished by day 28.^30^ Likewise, a phase 2b study in Japan involving participants with TRD indicated no significant difference between the esketamine and placebo groups at day 28.^31^ In this study, the mean reduction in MADRS score from baseline to 4 weeks was significantly higher in participants treated with esketamine, which contradicts the findings of previous studies showing a loss of efficacy within 4 weeks. This indicates that a single subanesthetic dose of esketamine has a positive effect on oral antidepressants in patients with FAD, since the expected spillover efficacy was aided by oral antidepressants. Furthermore, this effect could last for an extended period, as the mean reduction in MADRS score from 4 to 6 weeks in the esketamine-treated group remained significantly higher than in the control group. This interesting phenomenon suggests that a subanesthetic dose of esketamine could boost the efficacy of antidepressants, therefore providing preliminary evidence to confirm our hypothesis that esketamine therapy is a promising strategy for the treatment of FAD. The simplicity and high success rate of esketamine therapy would allow patients to continue using their current antidepressant regimen and avoid adverse effects associated with changes in the medication regimen, for example, unanticipated adverse effects and a reduced willingness to seek treatment due to repeated attempts.

The mechanism of ketamine and its enantiomers in exerting antidepressant effects is unclear, and few studies have reported the underlying interaction between ketamine and antidepressants. A study by Dean et al^32^ summarized the differences in efficacy between ketamine and other glutamate receptor modulators in the treatment of MDD, reporting that ketamine had higher response efficacy compared with midazolam at 24 hours, with very low–certainty evidence at 1 and 2 weeks, and no significant difference was observed at 4 weeks.^32^ In our study, the rates of response at 1, 2, 4, and 6 weeks were significantly higher in the esketamine-treated group compared with the midazolam control group. A study by Hu et al^33^ found that a single subanesthetic dose of ketamine plus newly initiated escitalopram was associated with significantly lower MADRS scores from 2 hours to 2 weeks. However, the mean reduction in MADRS score from baseline at each visit was significantly higher among participants treated with esketamine than among those treated with midazolam. The possible reasons for these inconsistencies could be the different types of participants and the unknown interaction of esketamine and antidepressants.^33^ Several reports have suggested that FAD may result from an evolving drug sensitization that causes a pharmacokinetic or pharmacodynamic tolerance, or both, to the previously effective concentration or drug actions.^34,35^ For patients with FAD receiving the same drug with a loss of efficacy, the underlying mechanism of ketamine’s effect on antidepressants might involve some unidentified interaction with antidepressants to alter antidepressant sensitization and improve tolerance to antidepressants. Although there is no direct evidence to suggest that ketamine and antidepressants interact, ketamine may act on the same receptors, with the most evidence for serotonin (eg, 5-hydroxytryptophan).^26^

Additionally, ketamine can alleviate depressive symptoms by restoring the lost prefrontal synaptic spines, which may partially explain the sustained antidepressant effects observed in our study.^36^ By modifying the structure of individual nervous system, ketamine may allow patients to restore antidepressant sensitivity. Based on our findings, it is hypothesized that ketamine or its enantiomer also exerts antidepressant effects via a reticular receptor-associated system involving several antidepressant receptors, which may include interaction with multiple antidepressant receptors.

### Limitations

This study has several limitations. First, symptom fluctuation was evaluated retrospectively using the VAS tool, and the definition of FAD was empirical without a reference standard, which may have introduced bias and affected data accuracy. However, proposing a definition of FAD was fundamental to the needs of clinical practice. An improved definition of FAD in the future requires adequate support of higher-quality evidence. Additionally, another potential weakness of the study was its small sample size in a single center, which may limit the generalizability of the results. What is more, including patients with first-episode depression and patients with recurrent depression in the sample may limit the internal and external validity. Furthermore, collecting data during the COVID-19 pandemic may have impacted the results of this study. Additionally, a significant difference in dissociation levels at 40 minutes was observed and blinding assessment was lacking, which could potentially affect the integrity of blinding.

## Conclusion

This double-blind randomized clinical trial found that a single subanesthetic dose of esketamine could boost the efficacy of oral antidepressants in the treatment of FAD with a good safety profile. Future larger trials with longer follow-up are required to confirm these findings.

## Notes

Supplement 1.
Trial Protocol and Statistical Analysis Plan

Supplement 2. **eTable 1.** Rates of Remission at Different Visits by Intervention and Control Group **eFigure.** The Least-Square Mean Reduction of MADRS Scores From Baseline to 6 Weeks at Each Visit by Intervention and Control Group **eTable 2.** Reduction of CGI-S Score From Baseline at Different Visits by Intervention and Control Group

Supplement 3.
Data Sharing Statement
