# Supplementary material for: Esketamine vs Midazolam in Boosting the Efficacy of Oral Antidepressants for Major Depressive Disorder: A Pilot Randomized Clinical Trial
Source: JAMA Netw Open. 2023 Aug 14;6(8):e2328817. doi: 10.1001/jamanetworkopen.2023.28817 (PMC10425830; doi:10.1001/jamanetworkopen.2023.28817)
Supplement: Supplement 1. — Trial Protocol and Statistical Analysis Plan [file jamanetwopen-e2328817-s001.pdf]

Study Number: 2021, No. (012)

# Efficacy and Safety of Single Subanesthetic S-ketamine in Boosting Efficacy of Oral Antidepressants for Major Depressive Disorder: A Randomized Double-Blind Midazolam-Controlled Pilot Trial

Final IRB Approved Protocol



| **Principle Invesitigator** |
|---|
| Gang Wang, MD, PhD<br>Professor of Medicine<br>Beijing Key Laboratory of Mental Disorders, National Clinical Research Center for Mental Disorders & National Center for Mental Disorders, Beijing Anding Hospital, Capital Medical University, 5 Ankang Hutong, Beijing 100032, China<br>Advanced Innovation Center for Human Brain Protection, Capital Medical University, 10 You’an Gate Waixi 1st Alley, Beijing 100069, China<br>gangwangdoc@ccmu.edu.cn |
| **Lead Statistician** |
| Haibo Wang, PhD<br>Peking University Clinical Research Institute, Peking University First Hospital, 38 Xueyuan St, Beijing 100191, China |

# Table of Contents


**Synopsis and Study Schema** ... 1

**Abbreviations** ... 4

1. Study Objective ... 5
2. Study Design ... 5
3. Study Participants ... 7
4. Randomization and Blinding ... 8
5. Study Treatments ... 8
6. Administration of Study Drug ... 10
7. Concomitant Medications and Treatments ... 10
8. Screening, Baseline Assessment and Follow-up ... 10
9. Study Evaluations and Endpoints ... 15
10. Subject Completion/Withdrawal ... 17
11. Statistical Methods ... 18
12. Adverse Event Reporting ... 19
13. Risk Assessment and Risk Management Plan ... 20
14. Emergency Unblinding ... 22
15. Ethical Principles ... 22
16. Data Management ... 23
17. Data Quality Assuarance/Quality Control ... 24
18. Third Party Audits ... 25
19. Data Confidentiality ... 25
20. References ... 27
21. Appendix ... 28

# Synopsis and Study Schema

| | |
|---|---|
| Study Title | Efficacy and Safety of Single Subanesthetic S-ketamine in Boosting Efficacy of Oral Antidepressants for Major Depressive Disorder: A Randomized Double-Blind Midazolam-Controlled Pilot Trial |
| Primary Objective | To assess the efficacy and safety of a single subanesthetic dose of S-ketamine in boosting efficacy of oral antidepressants for treating **F**luctuating symptoms occurring during adequate **A**ntidepressant treatment (ADT) in patients with major **D**epressice disorder (FAD) |
| Study Design | Single-center, double-blind, randomized midazolam-controlled pilot trial |
| Research Center | Beijing Anding Hospital, Capital Medical University |
| Number of Patients | 30 |
| Study population | ●Inclusion criteria:<br>① Outpatients/inpatients aged 18 to 60 years (inclusive), regardless of gender;<br>② Diagnosed major depressive disorder (MDD) according to Diagnostic and Statistical Manual of Mental Disorders, Fifth Edition (DSM-5) (confirmed by Mini International Neuropsychiatric Interview, Mandarin for China Translation Version 7.0.2);<br>③ Patients with fluctuating symptoms (VAS-2 score ⩽ 5) after symptom relief and stabilization (VAS-1 score ⩾ 8) on a single antidepressant treatment in the most recent episode, without any adjustments in the kind and dosage of the antidepressant; and the duration of drug interruption during this period was less than 7 days;<br>④ HAMD-17 score ⩾ 14 at screening;<br>⑤ The current dosage must meet the minimum dosage acquired in this study as determined by the Antidepressant Treatment History Form: Short Form (ATHF-SF) [see Appendix] or the minimum therapeutic dose as determined by the corresponding drug's instructions (for those drugs of which manufacturers not included in the ATHF-SF);<br>⑥ Able to understand and comply with study protocol;<br>⑦ Willing to participate and provide written informed consent.<br><br>●Exclusion criteria: |

| | |
|---|---|
| | Those presenting any of the following will be excluded from this study.<br>① Presence of a history of treatment-resistant depression (TRD), schizophrenia, bipolar disorder and obsessive-compulsive disorder;<br>② Persons with a psychotic disorder associated with other diseases;<br>③ Currently with psychotic features;<br>④ History of severe drug allergy or hypersensitivity reaction;<br>⑤ Women who are pregnant or breastfeeding;<br>⑥ "Yes" to item 4 or 5 of the Columbia-Suicide Severity Rating Scale (C-SSRS) "Suicidal Ideation" during the recent episode; or had suicidal behavior during the recent depressive episode according to the C-SSRS "Suicidal Behavior"; or was at serious risk for suicide according to the investigator's judgment;<br>⑦ Presence of a history of respiratory disease, such as chronic obstructive pulmonary disease, chronic bronchitis, bronchiectasis, etc.;<br>⑧ Presence of a history of cardiovascular disease, such as hypertension, various types of angina pectoris, myocardial infarction, etc.;<br>⑨ Alanine aminotransferase (ALT) or aspartate aminotransferase (AST) or gamma-glutamyl transferase (GGT) above the Upper Limit of Normal (ULN), or total bilirubin above the ULN, where the above abnormalities are clinically significant and assessed by the investigator to be dangerous participating;<br>⑩ Abnormal testing results with clinical significance for free triiodothyronine (FT3), free thyroxine (FT4) or thyroid stimulating hormone (TSH);<br>⑪ Contraindications to the use of S-ketamine in the presence of severe risk of elevated blood pressure or intracranial pressure, resting systolic/diastolic blood pressure above 180/100 mmHg, pre-eclampsia and eclampsia, hyperthyroidism, ischemic heart disease;<br>⑫ Presence of contraindications to the use of midazolam such as hypersensitivity to midazolam or any excipient of midazolam, acute angle-closure glaucoma, and untreated open-angle glaucoma;<br>⑬ Treatment history of electroconvulsive therapy within the past 3 months;<br>⑭ Presence of a history of ketamine or S-ketamine or its isomers exposure; |

| | |
|---|---|
| | ⑮ Presence of a history of substance abuse, drug (e.g., methamphetamine, etc.) use;<br>⑯ Abnormal and clinically significant findings during the screening period;<br>⑰ Those who screen positive for novel coronavirus nucleic acid;<br>⑱ There are other conditions that, in the opinion of the investigator, are inappropriate for participation in this trial. |
| Estimated duration of study | 1 year (enrollment and follow-up) |
| Follow-up plan | Baseline, 40 minutes, 2, 4 and 24 hours, 1, 2, 4 and 6 weeks after infusion |
| Interventions | All participants in the S-ketamine-treated group received single S-ketamine i.v. at 0.5 mg/kg within 40 minutes. The participants in the midazolam-treated group received single midazolam i.v. at 0.045 mg/kg within 40 minutes. |
| Outcomes | Primary outcome<br>Rate of response at 2 weeks, response was defined by 50% reduction of Montgomery-Åsberg Depression Rating Scale (MADRS).<br>Secondary outcomes<br>Rate of response at 6 weeks, rates of remission at 2 and 6 weeks, change in MADRS and Clinical Global Impression (CGI) score from baseline to 6 weeks, remission was defined by MADRS score of less than or equal to 10.<br>Safety assessments<br>General side effects, psychotogenic effects, dissociative effects, manic symptoms, and suicide risk. |

# Abbreviations

| | |
|---|---|
| ADT | Antidepressant treatment |
| ALT | Alanine Aminotransferase |
| AST | Aspartate Aminotransferase |
| ATHF-SF | Antidepressant Treatment History Form-Short Form |
| BMI | Body Mass Index |
| BPRS-4 | Four-item Positive Symptom Subscale of the Brief Psychiatric Rating Scale |
| CADSS | Clinician-administered Dissociative States Scale |
| CGI | Clinical Global Impression |
| CGI-EI | Clinical Global Impression-Efficacy Index |
| CGI-S | Clinical Global Impression-Severity |
| CGI-I | Clinical Global Impression-Improvement |
| C-SSRS | Columbia Suicide Severity Rating Scale |
| DSM-5 | Diagnostic and Statistical Manual of Mental Disorders (5th Edition) |
| ECT | Electroconvulsive Therapy |
| FAD | **F**luctuating symptoms (re-emergence of initial symptoms/episode or onset of a new episode) occurrs during adequate **A**ntidepressant treatment (ADT) in patients with M**D**D |
| FAS | Full Analysis Set |
| FIBSER | Frequency, Intensity, and Burden of Side Effects Rating |
| FT3 | Free Triiodothyronine |
| FT4 | Free Thyroxine |
| GGT | Gamma-glutamyl Transferase |
| MADRS | Montgomery-Åsberg Depression Rating Scale |
| MDD | Major Depressive Disorder |
| MECT | Modified Electroconvulsive Therapy |
| M.I.N.I 7.0 | Mini-International Neuropsychiatric Interview 7.0 |
| NMDA | N-methyl-D-aspartic Acid |
| PPS | Per Protocol Set |
| $SpO_2$ | oxygen saturation |
| SS | Safety Set |
| TSH | Thyrotropin |
| UKU | Udvalg for Kliniske Undersogelser |
| ULN | Upper Limit of Normal |
| VAS | Visual Analogue Scale/Score |
| VAS-1 | Visual Analogue Scale/Score-1 |
| VAS-2 | Visual Analogue Scale/Score-2 |
| YMRS | Young manic rating scale |

# 1. Study Objective

To assess the efficacy and safety of a single subanesthetic dose of S-ketamine in boosting efficacy of oral antidepressants for treating **F**luctuating symptoms occurring during adequate **A**ntidepressant treatment (ADT) in patients with M**D**D (FAD).

# 2. Study Design

This study is a single-center, double-blind, randomized controlled pilot trial with balanced randomization (1:1) conducted at Beijing Anding Hospital, Capital Medical University. All participants with FAD will be randomly assigned to receive either a subanesthetic dose of S-ketamine or midazolam.

All subjects in 2 groups will maintain their original drug regimen throughout the whole study.

**Figure 1.** demonstrates the study process for both groups.

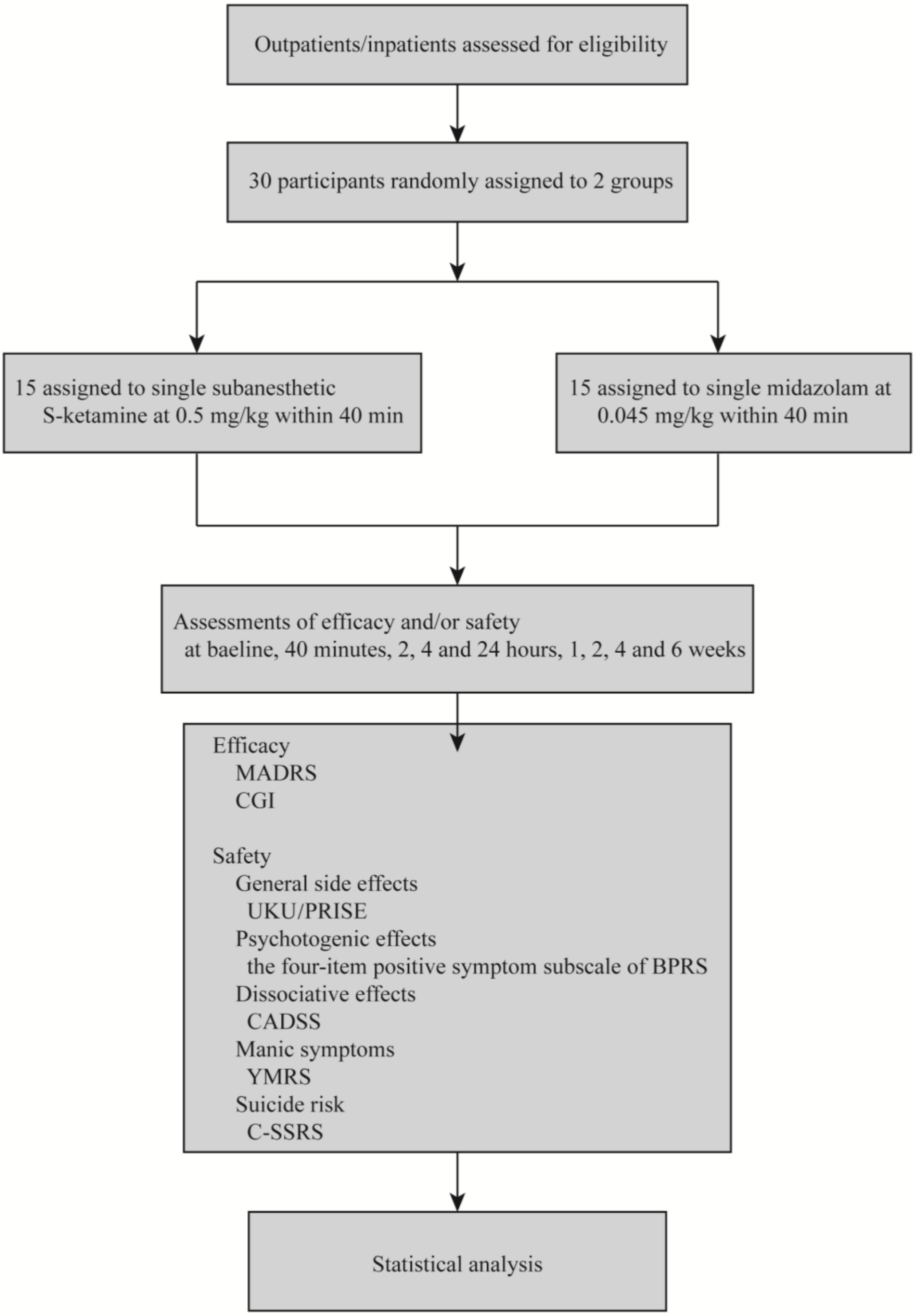


**Figure 1. Study Process**

# 3. Study Participants

## 3.1 Inclusion criteria

① Outpatients/inpatients aged 18 to 60 years (inclusive), regardless of gender;

② Diagnosed major depressive disorder (MDD) according to Diagnostic and Statistical Manual of Mental Disorders, Fifth Edition (DSM-5) (confirmed by Mini International Neuropsychiatric Interview, Mandarin for China Translation Version 7.0.2);

③ Patients with fluctuating symptoms (VAS-2 score ⩽ 5) after symptom relief and stabilization (VAS-1 score ⩾ 8) on a single antidepressant treatment in the most recent episode, without any adjustments in the kind and dosage of the antidepressant; and the duration of drug interruption during this period was less than 7 days;

④ HAMD-17 score ⩾ 14 at screening;

⑤ The current dosage must meet the minimum dosage acquired in this study as determined by the Antidepressant Treatment History Form: Short Form (ATHF-SF) [see Appendix] or the minimum therapeutic dose as determined by the corresponding drug's instructions (for those drugs of which manufacturers not included in the ATHF-SF);

⑥ Able to understand and comply with study protocol;

⑦ Willing to participate and provide written informed consent.

## 3.2 Exclusion criteria

Those presenting any of the following will be excluded from this study.

① Presence of a history of treatment-resistant depression (TRD), schizophrenia, bipolar disorder and obsessive-compulsive disorder;

② Persons with a psychotic disorder associated with other diseases;

③ Currently with psychotic features;

④ History of severe drug allergy or hypersensitivity reaction;

⑤ Women who are pregnant or breastfeeding;

⑥ “Yes” to item 4 or 5 of the Columbia-Suicide Severity Rating Scale (C-SSRS) "Suicidal Ideation" during the recent episode; or had suicidal behavior during the recent depressive episode according to the C-SSRS "Suicidal Behavior"; or was at serious risk for suicide according to the investigator's judgment;

⑦ Presence of a history of respiratory disease, such as chronic obstructive pulmonary disease, chronic bronchitis, bronchiectasis, etc.;

⑧ Presence of a history of cardiovascular disease, such as hypertension, various types of angina pectoris, myocardial infarction, etc.;

⑨ Alanine aminotransferase (ALT) or aspartate aminotransferase (AST) or gamma-glutamyl transferase (GGT) above the Upper Limit of Normal (ULN), or total bilirubin above the ULN, where the above abnormalities are clinically significant and assessed by the investigator to be dangerous participating;

⑩ Abnormal and clinically significant results of free triiodothyronine (FT3) or free thyroxine (FT4) or thyroid stimulating hormone (TSH) tests;

⑪ Contraindications to the use of S-ketamine in the presence of severe risk of elevated blood pressure or intracranial pressure, resting systolic/diastolic blood pressure above 180/100 mmHg, pre-eclampsia and eclampsia, hyperthyroidism, ischemic heart disease;

⑫ Presence of contraindications to the use of midazolam such as hypersensitivity to midazolam or any excipient of midazolam, acute angle-closure glaucoma, and untreated open-angle glaucoma;

⑬ Treatment history of electroconvulsive therapy within the past 3 months;

⑭ Presence of a history of ketamine or S-ketamine or its isomers exposure;

⑮ Presence of a history of substance abuse, drug (e.g., methamphetamine, etc.) use;

⑯ Abnormal and clinically significant findings during the screening period;

⑰ Those who screen positive for novel coronavirus nucleic acid;

⑱ There are other conditions that, in the opinion of the investigator, are inappropriate for participation in this trial.

## 4. Randomization and Blinding

Eligible patients were randomly assigned in a 1:1 ratio to receive either single S-ketamine i.v. or single midazolam i.v. for the same duration of 40 minutes. Randomization was performed using a computer-generated block randomization scheme (block size, 4) using SAS 9.4 (SAS Institute Inc., Cary, NC, USA) by a statistician who is not involved in the study. The generated randomization sequence was concealed in an opaque envelope. The independent assistant who is not involved in the study will be responsible for packing and labeling the prescribed medication. Study investigators, staff, participants, and all outcome assessors were blinded with respect to treatment assignment.

## 5. Study Treatments

The subjects will be admitted to the research ward specific to clinical trials one day before the day of infusion. After 6-hour preoperative fasting and water deprivation, the prescribed doses of drugs will be administered intravenously on the morning of the infusion day to the subjects. All subjects assigned to the trial group (S-ketamine-treated group) will receive single S-ketamine i.v. at 0.5 mg/kg within 40 minutes. The participants assigned to the control group (midazolam-treated group) will receive single midazolam i.v. at 0.045 mg/kg within 40 minutes. Both medications will be dissolved in 50 ml normal saline and administered for 40 minutes using a syringe pump. Safety checks will be performed before, during and after infusion in strict accordance with the safety check procedure of this study to ensure the safety of the subjects. During hospitalization before the subject's discharge, the subject should comply with the relevant ward-management rules of this study including maintaining a uniform light diet, and they are not allowed to enter or leave the ward at will without permission. On the morning of the next day of infusion, the subjects will be discharged after completing the visit at 24 hours.

Investigators are requested to carefully complete a preparation and safety check procedure to ensure the subject's safety.

- Pre-infusion preparation includes:

(1) The investigator verifies that the label of the drug is correct, that it is accurately dispensed, and that the blinding is complete;

(2) The principal investigator and the anesthesiologist rehearse in advance the emergency unblinding and adverse event response protocol flow;

(3) Reconfirm that the subject has no history of S-ketamine allergy, ketamine allergy, or midazolam allergy;

(4) Reconfirm the presence of systemic diseases such as hyperthyroidism and cardiovascular disease.

- Pre-, during and post-infusion safety checks include:

(1) Vital signs (within 30 minutes before infusion and 2, 4 and 8 hours after infusion, time window ±10 minutes after infusion): body temperature, respiration rate (ECG monitoring and manual measurement), heart rate, blood pressure;

(2) Oxygen saturation ($SpO_2$, time window as for vital signs);

(3) ECG monitoring (30 minutes before to 3 hours after infusion);

(4) 12-lead electrocardiogram (8 hours ± 1 hour after infusion);

(5) Collect information on concomitant medications and adverse events.

- Additionally, all subjects are requested to complete a vunluntary safety check process at 6 weeks (follow-up endpoint) at the end of the study, which specifically includes:

(1) Vital signs examination: body temperature, respiration rate, heart rate, and blood pressure;

(2) Laboratory tests (complete blood count, comprehensive metabolic panel, thyroid function, coagulation, and routine urinalysis);
(3) 12-lead electrocardiogram.

# 6. Administration of Study Drug

The management of S-ketamine and midazolam used in the study is in accordance with the "Management System for the Use of Narcotic and Class I Psychotropic Substances in Research at Beijing Anding Hospital".

# 7. Concomitant Medications and Treatments

During this trial, subjects will be requested to maintain their original medications. In principle, any other drugs are prohibited. Any drugs used during the trial other than those permitted in the trial should be recorded in detail in the CRF, and the record should include: drug name, dose, date of administration and indication for use. Strictly prohibited drugs include various N-methyl-D-aspartate receptor antagonists (e.g., meperidine, scopolamine, etc.), etc.

# 8. Screening, Baseline Assessment and Follow-up

Prior to conducting any study procedures, the investigator (or designated study personnel) will review and explain the written informed consent form (ICF) to each subject. After signing the ICF, the subject will be screened within 7 days to determine eligibility for study participation. The course of this study is specifically divided into three phases in total: screening phase (Day -7 – Day -1), baseline phase (Day 0), and follow-up phase (40 minutes, 2, 4 and 24 hours, 1, 2, 4 and 6 weeks after infusion). See **TIME AND EVENTS SCHEDULE** for detail.

## 8.1 Screening Phase (Day-7 - Day-1)

Subjects will be requested to voluntarily sign the ICF before participating in this trial. Screening will be conducted from Day -7 to Day -1 and subjects who meet all inclusion criteria and do not meet any of the exclusion criteria are allowed to participate in the trial.

All subjects will be given a unique subject code after signing the ICF. In this study, the subject code is a subject identifier, generated from the project abbreviation and the screening sequence number. The first 3 letters are the project abbreviation (EKD), and the last 2 digits are the sequence number

of the subject's entry into this study center for screening. For example: EKD01. During the screening interview, the investigator will collect all necessary information according to the protocol, including:

(1) Diagnosis confirmation:

*Mini-International Neuropsychiatric Interview* (MINI) was used in this study to confirm the diagnosis. MINI was first designed by Professors Sheehan and Lecrubier as a diagnostic instrument for *Diagnostic and Statistical Manual of Mental Disorders: 4th Edition (DSM-IV)* and *International Statistical Classification of Disease (DSM-10)*. The study used the latest version 7.0 based on *Diagnostic and Statistical Manual of Mental Disorders: 5th Edition (DSM-5)*.

(2) Symptom severity assessment:

Visual Analogue Scale/Score (VAS): The basic approach is to use a sliding scale between "0" and "10" segments. In this study, "0" means "seeing my normal state as a reference, my state is literally horrible during this period" and "10" means "seeing my normal state as a reference, my state is quite good (close to my normal state) during this period". The same scale was used for question 1: "How did you feel about your state during the period when your condition was stable, under the treatment of your current medication? "(VAS-1); Question 2: "How do you feel about your state during the recent period when you are not feeling well, under the treatment of your current medication"(VAS-2). The patient slides the scale to the appropriate position according to the subjective assessment of the degree of symptom fluctuation.

17-item Hamilton Rating Scale for Depression (HAMD-17): A scale developed by Hamilton in 1960 to assess the severity of depressive symptoms, with 17 items, the higher the total score, the more severe the depressive symptoms.

(3) General demographic information, including but not limited to:

date of birth, gender, education level, etc.; medical history: history of physical illness, history of psychiatric illness, family history of psychiatric illness.

(4) Clinical characteristics, including but not limited to:

age at onset of MDD, total course of MDD, number of MDD episodes, duration of recent episode, duration of ADT for recent episode, type and dosage of current antidepressant, duration of ongoing ADT with current antidepressant for recent episode, duration of maintaining stability after symptom relief with current antidepressant, duration of fluctuating symptoms with current antidepressant.

*Note: information on clinical characteristics was acquired mostly from patients, and reviewed and confirmed by the investigator based on medical records provided by patients and/or persuasive evidence from referring physicians.*

(5) Physical examination:

general appearance, skin mucosa, lymph nodes, head and neck, chest, abdomen, musculoskeletal, nervous system, height, weight, BMI.

(6) Vital signs examination:

temperature, respiration rate, heart rate, blood pressure.

(7) Urine drug testing.

(8) Infectious diseases screening:

hepatitis B surface antigen, hepatitis C antibody, HIV antibody, syphilis antibody, novel coronavirus nucleic acid.

(9) Laboratory tests:

complete blood count, comprehensive metabolic panel, thyroid function, coagulation, and routine urinalysis.

(10) 12-lead electrocardiogram and electroencephalogram.

(11) Suicide risk assessment:

Columbia-Suicide Severity Rating Scale (C-SSRS): The C-SSRS is a multidimensional instrument used to assess suicidal ideation, intensity of ideation, and suicidal behavior, and is scored by clinicians based on patient’s responses to questions.

(12) Concomitant medications and adverse events.

If the subject is able to present the results of infectious disease screening (excluding the novel coronavirus nucleic acid) within 1 week prior to the date of informed consent, and the study center can trace the results, the investigator may decide, based on the subject's condition, not to retest the relevant tests and use the test results presented by the subject as the basis for judgment; if the subject is unable to present the results of infectious disease testing within 1 week prior to the date of informed consent, or the study center cannot trace the results, the investigator should ask the subject to complete the relevant tests as soon as possible.

## 8.2 Baseline Phase (Day 0, within 24 hours prior to infusion)

(1) Rechecking patient’s eligibility to this study.

(2) Vital signs examination: body temperature, respiration rate, heart rate, blood pressure.

(3) Review of laboratory test results during the screening phase.

(4) 12-lead electrocardiogram.

(5) Assessments on efficacy and safety (Refer to the section of Follow-up Phase).

(6) Concomitant medications and adverse events.

## 8.3 Follow-up Phase (40 minutes, 2, 4 and 24 hours, 1, 2, 4 and 6 weeks after infusion)

Efficacy will be assessed at baseline (Day 0, within 24 hours prior to infusion), 40 minutes, 2, 4 and 24 hours, 1, 2, 4 and 6 weeks after infusion using MADRS (4-hour and 24-hour post-infusion visits using SIGMA 4-hour version and SIGMA 24-hour version, respectively) and CGI; side effects will be assessed using YMRS, BPRS-4, CADSS, and UKU/FIBSER; and the C-SSRS is used to assess suicide risk. At each visit, information on physical examination, vital signs, concomitant medications and adverse events will also be collected. See details in **Section 9. Study Evaluations**.

In this study, 2 raters who are qualified by consistent training are responsible for completing clinician-administered assessments and explaining patient-reported outcomes assessments to subjects at each visit.

## TIME AND EVENTS SCHEDULE

| Visit timepoints<br>Evaluation content | Screening Phase<br>Day -7 - Day -1 | Baseline Phase<br>Day 0<br>Within 24h before infusion | Follow-up phase | | | | | | | |
|---|---|---|---|---|---|---|---|---|---|---|
| | | | Visit 1<br>40min (±10min) | Visit 2<br>2h | Visit 3<br>4h | Visit 4<br>24h | Visit 5[a]<br>Day 7 | Visit 6[a]<br>Week 2 (±2 days) | Visit 7[a]<br>Week 4 (±2 days) | Visit 8[a]<br>Week 6 (±2 days) |
| Informed Consent Form | × | | | | | | | | | |
| M.I.N.I 7.0 | × | | | | | | | | | |
| General, demographic & clinical characteristics | × | | | | | | | | | |
| Medical history | × | | | | | | | | | |
| Vital signs | × | × | × | × | × | × | ×[b] | ×[b] | ×[b] | ×[b] |
| VAS-1& VAS-2 | × | | | | | | | | | |
| Infectious disease screening[c] | × | | | | | | | | | |
| Laboratory tests[d] | × | | | | | | | | | ×[b] |
| Urine drug testing | × | | | | | | | | | |
| Eligibility | × | | | | | | | | | |
| Concomitant medications | × | × | × | × | × | × | × | × | × | × |
| Adverse events | × | × | × | × | × | × | × | × | × | × |
| 12-lead electrocardiogram | × | × | | | | | | | | ×[b] |
| Electroencephalogram | × | | | | | | | | | |
| HAMD-17 | × | | | | | | | | | |
| MADRS | | × | | × | × | × | × | × | × | × |
| CGI | | × | | × | × | × | × | × | × | × |
| YMRS | | × | | | | × | × | × | × | × |
| BPRS-4 | | × | × | × | × | × | × | × | × | × |
| CADSS | | × | × | × | × | × | | | | |
| UKU/FIBSER | | × | | | | × | × | × | × | × |
| C-SSRS | × | × | | | | × | × | × | × | × |

[a]with telephone interview acceptable.

[b]may not be able to be collected in the case of telephone interview.

[c](Hepatitis B, Hepatitis C, Syphilis, HIV, novel coronavirus nucleic acid); [d](complete blood count, comprehensive metabolic panel, thyroid function, coagulation, and routine urinalysis)

# 9. Study Evaluations and Endpoints

## 9.1 Study Evaluations

### 9.1.1 Efficacy

#### 9.1.1.1 Primary

Montgomery-Asberg depression rating scale (MADRS): The primary efficacy evaluation will be the MADRS total score.The MADRS is a clinician-rated scale designed to measure depression severity and detects changes due to antidepressant treatment. The test consists of 10 items, each of which is scored from 0 (item not present or normal) to 6 (severe or continuous presence of the symptoms), for a total possible score of 60. Higher scores represent a more severe condition. The MADRS evaluates apparent sadness, reported sadness, inner tension, sleep, appetite, concentration, lassitude, interest level, pessimistic thoughts, and suicidal thoughts. The test exhibits high inter-rater reliability.

The structured interview guide for the Montgomery Asberg Depression Rating Scale (SIGMA) will be used on the visits of 4-hour and 24-hour with its corresponding version. Using structured interview guides have previously been shown to increase the reliability of given scales.The SIGMA used in this study was approved by the copyright holder, Mr. Adam Hirschberg from Cambridge University Press. Written permission was presented in Supplementary.

The SIGMA 4-hour version used for this study was translated into Chinese from the original language (English) by the first author who is a native Chinese speaker and fluent in English, and then was checked for accuracy in meaning and cultural sensitivity by an expert with her research interest mainly lies in the treatment of MDD who is fluent in Chinese and English. Discrepancies were discussed and resolved by joint agreement of both translators.

#### 9.1.1.2 Secondary

Clinical Global Impression (CGI): The CGI assessment, a widely used assessment tool in psychiatry for clinical trials, is a 3-item observer-rated scale that measures illness severity (SI), global improvement (GI) and therapeutic effects (EI). CGI-I is a seven point scale requiring clinicians to assess how much the patient's illness has improved or worsened relative to a baseline state at the beginning of the intervention. On the 7 point scale, 1 point was used for very much improved; 2 points for much improved; 3 for minimally improved; 4 for no change; 5 for minimally worse; 6 for much worse; and 7 for very much worse. The CGI-S is a clinician-rated scale that is designed to rate the

severity of the subject's illness at the time of assessment, relative to the clinician's past experience with subjects who have the same diagnosis and improvement with treatment. Considering total clinical experience, a subject is assessed on severity of mental illness at the time of rating according to: 0= not assessed; 1=normal (not at all ill); 2=borderline mentally ill; 3=mildly ill; 4=moderately ill; 5=markedly ill; 6=severely ill; 7=among the most extremely ill patients. EI ratings take account of both therapeutic efficacy and treatment-related adverse events and range from 0 (marked improvement with no side-effects) and 4 (unchanged or worse with side-effects outweighing the therapeutic effects).

### 9.1.3 Safety

#### 9.1.3.1 General side effects

Udvalg for Kliniske Undersogelser (UKU): The UKU consists of three components: an individual symptom assessment scale, an overall assessment scale, and the effect of the side effect on subsequent treatment. 48 symptoms were included and divided into four categories: psychiatric, neurological, autonomic, and other, the latter were mainly skin and sexual related side effects. A 4-point scale was used to score the side effects, taking into account the correlation between medication and side effects.
Frequency, Intensity, and Burden of Side Effects Rating (FIBSER): Developed by Rush et al. for the STAR*D study, the FIBSER consists of three questions on a seven-point scale from 0-6, which assesses the frequency, intensity, and burden of side effects in general, with good reliability and validity.

#### 9.1.3.2 Psychiatric effects

Clinician Administered Dissociative States Scale (CADSS): CADSS is a common scale used by clinicians to assess dissociative symptoms. It consists of 27 items, including 19 subjective items and 8 objective items, rated on a 5-point scale from 0-4.
Young manic rating scale (YMRS): A symptom rating scale mainly used to assess manic symptoms and their severity, consisting of 11 items, among which, except for items 5, 6, 8 and 9 which are rated on a scale of 0-8, all other items are rated on a scale of 0-4, the higher the score, the more severe the symptoms.
Four-item Positive Symptom Subscale of the Brief Psychiatric Rating Scale: Developed by Overall and Gorham in 1962, BPRS is one of the most widely used psychiatric rating scales. The change of the total score before and after treatment can better reflect the efficacy of treatment. In the present

study, we chose a 4-item positive symptom subscale to assess 4 symptoms including conceptual disorganization, suspiciousness, unusual thought content and hallucinations, with reference to previous similar studies.

#### 9.1.3.3 Suicide risk assessment

Columbia-Suicide Severity Rating Scale (C-SSRS): The C-SSRS is a multidimensional instrument used to assess suicidal ideation, intensity of ideation, and suicidal behavior, and is scored by clinicians based on patient's responses to questions.

## 9.2 Study Endpoints

### 9.2.1 Primary

Rate of response at 2 weeks, response was defined by 50% reduction of Montgomery-Åsberg Depression Rating Scale (MADRS).

### 9.2.2 Secondary

Rate of response at 6 weeks, rates of remission at 2 and 6 weeks, change in MADRS and Clinical Global Impression (CGI) score from baseline to 6 weeks, remission was defined by MADRS score of less than or equal to 10.

### 9.2.3 Safety

General side effects, psychotogenic effects, dissociative effects, manic symptoms, and suicide risk.

# 10. Subject Completion/Withdrawal

## 10.1 Completion

A subject will be considered to have completed the treatment if (s)he has completed assessments at 24 hours of the follow-up phase. Subjects who prematurely discontinue study treatment for any reason before completion of the assessment at 24 hours will not be considered to have completed the treatment.

## 10.2 Withdrawal From the Study

A subject will be withdrawn from the study for any of the following reasons:

① Voluntary withdrawal: subjects may voluntarily withdraw from the study at any time by withdrawing informed consent form;

② Subjects who are assessed by the C-SSRS as having a high risk of suicide or who are considered having a high risk of suicide by the study investigator;

③ Other circumstances such as change in primary diagnosis that are judged by the investigator to be currently unsuitable for participation in the study;

④ Presense of serious adverse events;

⑤ Investigator's decision: including but not limited to poor adherence to the study, occurrence of conditions that meet the exclusion criteria, inability to cooperate with study procedures for objective reasons, etc., the investigator may ask the subject to withdraw from the study;

⑥ Pregnancy events that are not planned to be terminated; and

⑦ Lost of follow-up.

# 11. Statistical Methods

Analyses were done on an intention-to-treat basis, including all randomized patients who completed assessments at baseline and received study medication (full analysis set, FAS). Missing data of MADRS score was imputed using the last observation carried forward (LOCF).

Continuous variables was presented with mean (standard deviation), or median (interquartile range). Categorical variables was presented with the number and percentages. Chi-square tests, Fisher's exact test, independent-samples *t* test or Wilcoxon rank-sum test were used, as appropriate, to compare the differences of baseline variables between intervention and control group.

Treatment effects on primary outcome were evaluated using Chi-Squared test. Sensitivity analysis was also done for the primary outcome using the logistic regression model with baseline MADRS score and the number of MDD episodes as covariates.

The secondary outcomes - the reduction in MADRS score and CGI-S from baseline over time – were analyzed using independent-samples t tests or Wilcoxon rank-sum test. The sensitivity analysis was also performed using covariance (ANCOVA) model with baseline MADRS score and the number of MDD episodes as covariates. The rates of remission were compared using Chi-Squared test.

All tests of statistical significance were two-sided, and a *P* value of <0.05 was considered statistically significant. All the statistical analyses were performed with SAS statistical software, version 9.4 (SAS Institute, Cary, N.C.).

# 12. Adverse Event Reporting

## 12.1 Definition of adverse events (AE)

An adverse event (AE) is any adverse medical condition that occurs in a patient or clinical study subject following the use of a product that is not necessarily causally related to such treatment. Therefore, an AE can be any unintended adverse sign (including abnormal laboratory findings), symptom, or disease that is temporally related to the investigational product, but not necessarily to itself. Pre-existing disease that worsens during the study period will be reported as an AE.

- Mild: Discomfort but does not interfere with daily activities
- Moderate: Discomfort, reduced or impaired daily activities
- Severe: unable to work or perform daily activities

Adverse events will be collected from the time of signing the ICF and study screening operation until the end of the study. An adverse event record form should be completed faithfully during the trial to record the time of occurrence, severity, duration, measures taken, and regression of the adverse event. Adverse events should be recorded on the designated CRF adverse event form, with adequate description of the classification criteria for determining the relationship of the adverse event to the investigational drug.

## 12.2 Treatment-related adverse events

The relevance of AEs to treatment was assessed by the investigator. The investigator should make an assessment of the possible association between the adverse event, the study drug, and the concomitant drug, rated with reference to the following 5-level classification criteria.

- Definitely relevant: the reaction appears in a reasonable chronological order of administration, it is consistent with the type of reaction known for the suspected drug, it disappears after discontinuation of the suspected drug, and the patient's clinical state or other reasons are unlikely to produce the reaction.
- Likely to be related: the reaction appears in a reasonable chronological order of administration, is consistent with the type of reaction known for the suspected drug, the reaction is significantly reduced after discontinuation of the suspected drug, and the patient's clinical status or other reasons are unlikely to produce the reaction.
- Possibly related: the reaction occurs in a reasonable chronological order of administration, is consistent with the type of reaction known for the suspected drug, and may abate upon

discontinuation of the suspected drug, but the patient's clinical status or other reasons may also produce the reaction.

- Possibly unrelated: the reaction does not appear in a reasonable time sequence after administration, does not fit well with the type of reaction known for the suspected drug, the reaction does not abate after discontinuation of the suspected drug, the patient's clinical state or other causes may have produced the reaction, and the reaction abates after improvement of the disease state or elimination of other causes.
- Definitely unrelated: the reaction does not occur in a reasonable time sequence after administration, does not correspond to the type of reaction known for the suspected drug, the patient's clinical state or other causes can produce the reaction, and the reaction is reduced after the disease state improves or other causes are eliminated.

### 12.3 Serious adverse events (SAEs)

- A serious adverse event (SAE) is any event that suggests a significant hazard, contraindication to use, side effect, or requires attention. An AE is required at any dose that meets at least one of the following criteria.
- Fatal; [Note: death is an adverse event ending, not an event].
- Life-threatening [Note: the term "life-threatening" refers to an event that, at the time of occurrence, presents an immediate risk of death to the patient; it does not refer to an event that would hypothetically result in death if it were more severe].
- Hospitalization or extension of original hospital stay.
- Causing persistent or significant functional incompetence/disability.
- Congenital malformations/birth defects.
- Medically important or requires intervention to prevent one or more of these outcomes.

From the signing of the ICF (the start of study screening), any clinical AE that meets the criteria for a SAE that occurs during the course of the study (including long-term follow-up) must be reported to the IEC and medical administration within 1 working day of the investigator being informed of the SAE, and the investigator must complete the SAE report form, send it to the SAE administer in charge, and file it with the IEC of the research center.

## 13. Risk Assessment and Risk Management Plan

Since this study involves an intervention in the form of invasive manipulation, it may pose potential risks to the physical and mental health of the subjects. The investigators should provide medical care

to the subjects, achieve early identification, early management and safety of side effects, fully improve the risk management plan and ensure the safety of the subjects.

## 13.1 Risk Assessment

(1) The investigational drug in this study, S-ketamine, replaces the classical ketamine drug, which has been used in patients with major depressive disorder due to its rapid antidepressant effect, with a higher guarantee of safety and fewer side effects. However, there is still some risk of side effects such as psychiatric symptoms and a very low risk of allergic reactions. Prior to the start of the trial, the investigator is required to inform subjects that they can contact the investigator at any time if they have any questions about the study or if anything unusual occurs.

(2) In order to fully protect the rights of the subjects and ensure that their right to life and health is not violated, safety checks are strictly conducted at the necessary visits. The subject's suitability for further participation in the study will be actively assessed and confirmed at each visit throughout the study.

(3) If a subject experiences any physical or psychological discomfort related to this study during the course of participation in this study, the investigator will provide the necessary support services in the first instance.

## 13.2 Management Plan

(1) Subjects have the right to voluntarily withdraw or terminate participation at any time. If a subject is unable to continue to participate in the study, (s)he should contact the investigator and inform him or her of the current situation, and the investigator will take the necessary supportive measures according to the circumstances.

(2) If a subject is assessed at the follow-up visit to show a high level of suicide risk, (s)he is considered unable to continue to participate in the study and the investigator is required to notify the principal investigator at the first opportunity and inform the subject of his or her current condition. The subject will be terminated early and treatment will be selected based on his or her actual condition and wishes.

(3) Contingency plan for the risk of intravenous administration of S-ketamine.

Side effects to S-ketamine are usually dose- and frequency- dependent and are spontaneous and reversible. Common side effects include nausea, vomiting, tachycardia, increased blood pressure, and psychotic symptoms.

Respiratory depression or respiratory failure may result during high dose administration or rapid intravenous administration, and assisted artificial respiration is given until adequate spontaneous

breathing is restored. Since the possibility of respiratory depression cannot be completely ruled out, the research ward has been prepared with intubation and ventilation equipment and the appropriate resuscitation necessary equipment, and the necessary training has been provided to the personnel involved. Patients will also be monitored appropriately and continuously after the administration of the drug to ensure their safety.

Administration of benzodiazepines or antipsychotics, may reduce the psychiatric-like side effects of S-ketamine (e.g., agitation, delirium, dissociative symptoms, anxiety, babbling, confusion, etc.).

S-ketamine is metabolized in the liver and requires clearance by the liver to terminate clinical effects. Abnormalities in liver function tests associated with S-ketamine use have been reported, particularly with prolonged use (>3 days) or drug abuse. Prolonged duration of action may occur in patients with cirrhosis or other types of liver injury. In this trial, appropriate laboratory tests such as biochemistry rountine were performed during the screening phase to exclude those with significant abnormal liver function from enrollment.

(4) Contingency plan for the risk of intravenous midazolam administration.

Intravenous administration in adults may result in side effects, including

- Neurological system: excessive sedation (1.6%), headache (1.5%), drowsiness (1.2%)
- Digestive system: nausea (2.8%), vomiting (2.6%), congestion (2.6%)
- Skin: local symptoms (5.6%), painful injections (5%), sclerosis (1.7%), phlebitis (0.4%), congestion (2.6%).

Patients who experience these side effects should be treated symptomatically at first and referred to specialist care if necessary.

As with S-ketamine, midazolam, a benzodiazepine sleep-inducing agent, has CNS effects depending on the dose administered and may cause respiratory depression at high doses, which can be treated in the same way as S-ketamine induced respiratory depression or respiratory failure.

## 14. Emergency Unblinding

This is a double-blind, randomized, controlled study, and in the event of an emergency or a subject requiring resuscitation where it must be known what treatment the subject is receiving, the principal investigator will be notified by the first site investigator in the first instance, and approval will be granted for unblindling according to the procedures set forth in the study protocol. Once unblinding, the trial will be discontinued for that numbered case and the reason for discontinuation will be recorded on the CRF.

## 15. Ethical Principles

This clinical study must be conducted in accordance with the Declaration of Helsinki and the relevant clinical trial norms and regulations in China. Information including the study protocol and ICF must be submitted for approval by the Ethics Committee of Beijing Anding Hospital, Capital Medical University before the study can be started.

All subjects in both groups maintained the original medication regimen. The starting point for the use of S-ketamine as the trial drug was that previous studies have found that S-ketamine has a rapid antidepressant effect in patients with major depressive disorder with good safety profile, while studies on S-ketamine as a boost agent to oral antidepressants in the treatment of major depressive disorder have not been conducted. Therefore, the effecy for this use is unexplored and its efficacy remains unconformed. The design of this study guarantees to a certain extent a controlled difference in efficacy between the experimental and control groups to avoid too pronounced intervention effects or extremely poor efficacy that would violate ethical principles. The reason why this study select midazolam as a control drug: midazolam is a placebo that has no specific antidepressant effect but can induce the same transient psychoactive response as S-ketamine. On the one hand, the “unblinding damage” caused by the difference in psychoactive response between the two drugs was minimized. On the other hand, the possible effects of S-ketamine as an anesthetic agent to play its therapeutic role in the treatment of major depressive disorder were minimized, so as to balance the non-specific therapeutic benefits of the two groups.

If there is a need to make changes to the study protocol, ICF and other study documents during the study, they must be reported to the IEC for review and approval before implementation. If serious adverse events occur during the study, they should be reported to the IEC as required.

Prior to the start of screening, it is the responsibility of the investigator to provide subjects and their guardians with an objective and comprehensive description of the purpose, procedures, and possible benefits and risks of the study. Patients should be made aware of their right to decide whether to participate in the study and to request withdrawal from the study. An ICF signed by the patient must be obtained by the investigator prior to each patient's entry into the study and maintained as a study document.

Compliance with relevant regulations regarding personal information protection and privacy will be described in the ICF. In accordance with this instruction, patients authorize the collection, use and publication of their data by the investigator or other persons who need to be informed for the purposes of this study. Study data are to be kept strictly confidential in accordance with the appropriate national laws.

# 16. Data Management

A specific data management plan was developed for this study, and the data management task was carried out according to the data management plan specifications after the approval of the principal investigator. The specific data management tasks include authorization of data management personnel, creation of a paper-based database based on the original records of the study protocol, electronic data management using the data management software Epidata, and processing and archiving of data information.

(1) Design of the original record form: The paper version of the CRF was designed for data collection according to the requirements of the protocol, and the corresponding data collection guidelines were formed and finalized after review and approval by the principal investigator.

(2) Data verification: The data manager writes the data management plan based on the study protocol and study medical records, and finalizes it for use after review and approval by the principal investigator, and performs data management on the data based on the data management plan.

# 17. Data Quality Assuarance/Quality Control

The implementation, management, quality control, data management and statistical analysis of this trial were specifically undertaken and completed by Beijing Anding Hospital, Capital Medical University.

## 17.1 Protocol Amendments

The study protocol is written by the principal investigator based on a thorough review of the literature and is executed with the approval of the institutional ethics committee. No one who will have the right to modify this protocol without a formal amendment.

If the study protocol must be amended, the amendment and/or the new version of the protocol (amended protocol) must be submitted to the IEC for approval prior to implementation. In the case of a non-significant change, this change must be submitted to and approved in writing by the IEC in accordance with the relevant requirements. If the protocol amendment requires a change in the ICF of the research center, the IEC of the center must be notified. The application of the amended ICF must be approved in writing by the IEC.

## 17.2 Quality Management

A fixed quality control system was established for this trial, so that the study will follow the protocol and comply with relevant regulations and ensured that the study records and reports are accurate and

complete. If any inconsistency with the study protocol occurs during the trial, it should be reported in a timely manner and appropriate measures should be taken. Each phase of the study will be conducted in accordance with the standard operating procedures for clinical trial quality management and data quality assurance to ensure reliable data and correct study process.

## 17.3 Early Termination

If a subject is unable to continue to participate in the study, please contact the investigator promptly and inform him/her of the current situation.

## 17.4 Personnel Training

A study initiation meeting attended by study personnel will be held before the start of the study to conduct training on the GCP and study protocol, CRF completion and operational procedures to ensure that each personnel is familiar with the study protocol and procedures and that the study is conducted under uniform standards and requirements.

In this study, two investigators will be selected to be responsible for the follow-up of the subjects. Beijing Anding Hospital, Capital Medical University, will unify the training of the personnel as the research center and take the responsibility of supervising the good practice of the study protocol.

A paper version of the CRF form is used in this study, which was completed jointly by the designated personnel and the subject and should not be altered at will (in cases where alterations are necessary, the relevant instructions for this study regarding CRF alterations should be followed), and the original record form must be completed promptly and accurately for each case. Completed forms will be collected after confirmation by the CRC and sealed in a timely manner after review and confirmation.

# 18. Third Party Audits

All data of the study will be monitored by the competent unit. The data monitor will review the completeness and authenticity of the data against the original documents of the registry for all CRFs according to the monitoring plan.

All data corrections to the database will be made in an audit trail manner. The key variables identified for statistical analysis will be collated based on a 5% review at the end of registration to maintain an error rate of 0.05%.

# 19. Data Confidentiality

In strict accordance with GCP requirements, each clinical unit shall maintain all study data, including medical records, CRFs, and various original records (including all original, signed patient ICFs), for a period of 5 years. The principal investigator is responsible for the management of the data under seal, and any circumstances involving the use of the data must be reviewed by the principal investigator and submitted to the study sponsor for approval before implementation.

# 20. References

1. Alqahtani AM, Kumarappan C, Kumar V, et al. Understanding the genetic aspects of resistance to antidepressants treatment[J]. Eur Rev Med Pharmacol Sci, 2020, 24(14): 7784-7795. 10.26355/eurrev_202007_22281
2. Andrade C. Ketamine for Depression, 1: Clinical Summary of Issues Related to Efficacy, Adverse Effects, and Mechanism of Action[J]. J Clin Psychiatry, 2017, 78(4): e415-e419. 10.4088/JCP.17f11567
3. Andrade C. Ketamine for Depression, 4: In What Dose, at What Rate, by What Route, for How Long, and at What Frequency?[J]. J Clin Psychiatry, 2017, 78(7): e852-e857. 10.4088/JCP.17f11738
4. Chen MH, Li CT, Lin WC, et al. Persistent antidepressant effect of low-dose ketamine and activation in the supplementary motor area and anterior cingulate cortex in treatment-resistant depression: A randomized control study[J]. J Affect Disord, 2018, 225: 709-714. 10.1016/j.jad.2017.09.008
5. Cosci F, Mansueto G, Fava GA. Relapse prevention in recurrent major depressive disorder. A comparison of different treatment options based on clinical experience and a critical review of the literature[J]. Int J Psychiatry Clin Pract, 2020, 24(4): 341-348. 10.1080/13651501.2020.1779308
6. Deyama S, Bang E, Wohleb ES, et al. Role of Neuronal VEGF Signaling in the Prefrontal Cortex in the Rapid Antidepressant Effects of Ketamine[J]. Am J Psychiatry, 2019, 176(5): 388-400. 10.1176/appi.ajp.2018.17121368
7. Hu YD, Xiang YT, Fang JX, et al. Single i.v. ketamine augmentation of newly initiated escitalopram for major depression: results from a randomized, placebo-controlled 4-week study[J]. Psychol Med, 2016, 46(3): 623-635. 10.1017/s0033291715002159
8. Huang Y, Wang Y, Wang H, et al. Prevalence of mental disorders in China: a cross-sectional epidemiological study[J]. Lancet Psychiatry, 2019, 6(3): 211-224. 10.1016/s2215-0366(18)30511-x
9. Mocking RJT, Naviaux JC, Li K, et al. Metabolic features of recurrent major depressive disorder in remission, and the risk of future recurrence[J]. Transl Psychiatry, 2021, 11(1): 37. 10.1038/s41398-020-01182-w
10. Murrough JW, Abdallah CG, Mathew SJ. Targeting glutamate signalling in depression: progress and prospects[J]. Nat Rev Drug Discov, 2017, 16(7): 472-486. 10.1038/nrd.2017.16
11. Murrough JW, Iosifescu DV, Chang LC, et al. Antidepressant efficacy of ketamine in treatment-resistant major depression: a two-site randomized controlled trial[J]. Am J Psychiatry, 2013, 170(10): 1134-1142. 10.1176/appi.ajp.2013.13030392
12. Sackeim HA, Aaronson ST, Bunker MT, et al. The assessment of resistance to antidepressant treatment: Rationale for the Antidepressant Treatment History Form: Short Form (ATHF-SF)[J]. Journal of Psychiatric Research, 2019, 113: 125-136. https://doi.org/10.1016/j.jpsychires.2019.03.021
13. Singh JB, Fedgchin M, Daly E, et al. Intravenous Esketamine in Adult Treatment-Resistant Depression: A Double-Blind, Double-Randomization, Placebo-Controlled Study[J]. Biol Psychiatry, 2016, 80(6): 424-431. 10.1016/j.biopsych.2015.10.018
14. Smith K. Mental health: a world of depression[J]. Nature, 2014, 515(7526): 181. 10.1038/515180a
15. Su TP, Chen MH, Li CT, et al. Dose-Related Effects of Adjunctive Ketamine in Taiwanese Patients with Treatment-Resistant Depression[J]. Neuropsychopharmacology, 2017, 42(13): 2482-2492. 10.1038/npp.2017.94
16. Williams NR, Heifets BD, Blasey C, et al. Attenuation of Antidepressant Effects of Ketamine by Opioid Receptor Antagonism[J]. Am J Psychiatry, 2018, 175(12): 1205-1215. 10.1176/appi.ajp.2018.18020138
17. Williams NR, Schatzberg AF. NMDA antagonist treatment of depression[J]. Curr Opin Neurobiol, 2016, 36: 112-117. 10.1016/j.conb.2015.11.001
18. Yang Y, Cui Y, Sang K, et al. Ketamine blocks bursting in the lateral habenula to rapidly relieve depression[J]. Nature, 2018, 554(7692): 317-322. 10.1038/nature25509
19. Yiend J, Paykel E, Merritt R, et al. Long term outcome of primary care depression[J]. J Affect Disord, 2009, 118(1-3): 79-86. 10.1016/j.jad.2009.01.026
20. Zanos P, Moaddel R, Morris PJ, et al. NMDAR inhibition-independent antidepressant actions of ketamine metabolites[J]. Nature, 2016, 533(7604): 481-486. 10.1038/nature17998
21. Zeifman RJ, Singhal N, Dos Santos RG, et al. Rapid and sustained decreases in suicidality following a single dose of ayahuasca among individuals with recurrent major depressive disorder: results from an open-label trial[J]. Psychopharmacology (Berl), 2020. 10.1007/s00213-020-05692-9
22. Zheng W, Zhou Y-L, Liu W-J, et al. Rapid and longer-term antidepressant effects of repeated-dose intravenous ketamine for patients with unipolar and bipolar depression[J]. Journal of Psychiatric Research, 2018, 106: 61-68. https://doi.org/10.1016/j.jpsychires.2018.09.013

# 21. Appendix

## Antidepressant Treatment History Form-Short Form (excerpt)

| Medication | Brand Names | Minimum Oral Dose/day |
|---|---|---|
| **SSRIs** | | |
| Citalopram | Celexa, Cipramil | 20mg |
| Escitalopram | Lexapro, Cipralex, Sipralexa | 20mg |
| Fluvoxamine | Luvox (including CR), Floxyfral | 200mg |
| Fluoxetine | Prozac, Sarafem | 20mg |
| Paroxetine | Paxil, Pexeva, Brisdelle, Seroxat | 20mg |
| Paroxetine CR | Paxil CR | 25mg |
| Sertraline | Zoloft, Lustral, Serlain | 100mg |
| **SNRIs** | | |
| Desvenlafaxine | Pristiq, Khedezla, Desfax, Ellefore | 50mg |
| Duloxetine | Cymbalta, Irenka | 40mg |
| Levomilnacipran | Fetzima | 40mg |
| Venlafaxine | Effexor, Effexor XR | 150mg |
| **TCAs/Tetracyclines** | | |
| Amitriptyline | Elavil, Endep, Levate, Redomex | 200mg |
| Clomipramine | Anafranil, Clofranil | 200mg |
| Desipramine | Norpramine, Pertofrane | 200mg |
| Doxepin | Sinequan, Adapin, Silenor | 200mg |
| Imipramine | Tofranil | 200mg |
| Maprotilene | Ludiomil | 200mg |
| Mianserin | Athymil, Bolvidon, Deprevon, Lerivon, Miansan, Serelan, Tetramide, Tolvin | 30mg |
| Nomifensine | Merital, Alival | 200mg |
| Nortriptyline | Pamelor, Aventyl, Nortrilen | 75mg |
| Protriptyline | Vivactil | 40mg |
| Trimipramine | Surmontil, Rhotrimine, Stangyl | 200mg |
| **MAOIs** | | |
| Isocarboxazid | Marplan, Marplon, Enerzer | 40mg |
| Moclobemide | Amira, Aurorix, Clobemix , Depnil, Manerix | 300mg |
| Phenelzine | Nardil, Nardelzine | 60mg |
| Selegiline | Eldepryl, Zelapar | 40mg |
| Selegiline Patch | Emsam | 6mg |
| Tranylcypromine | Parnate | 40mg |
| **Other** | | |
| Agomelatine | Melitor, Thymanax, Valdoxan | 25mg |
| Amoxapine | Asendin, Asendis, Defanyl, Demolox | 400mg |
| Bupropion | Wellbutrin (IR, SR & XL), Aplenzin, Forfivo XL | 300mg |
| Mirtazapine | Remeron, Remergon | 30mg |
| Nefazodone | Serzone | 300mg |
| Tianeptine | Coaxil, Salymbra, Stablon, Tatinol, Tianeurax, Zinosal | 37.5mg |
| Trazodone IR | Desyrel, Trazolan, Nestrolan | 400mg |
| Trazodone ER | Oleptro | 200mg |
| Vilazodone | Viibryd | 20mg |
| Vortioxetine | Brintellix, Trintellix | 20mg |

**Copy of written permission from Sir. Adam Hirschberg**

July 16, 2021

Dr. Chunfeng Xiao
Beijing Anding Hospital
Capital Medical University
Beijing, China
The National Clinical Research Center for Mental Disorders & Beijing Key Laboratory of Mental Disorders
5 Ankang Hutong
Beijing 100032
China

Dear Chunfeng Xiao,

**Use of the SIGMA (4-hour and 24-hour) in Single i.v. esketamine as boost agent to antidepressants in the treatment of major depressive disorder: A double-blind randomized placebo-controlled trial**

Thank you for your request to use copies of this rating scale in the above referenced study. I would like to confirm the following:

- Cambridge University Press hereby grants you permission to make up to 100 administrations of the SIGMA either electronically or in paper form.
-
- You may make copies of the scale in any format as required in order to do this.
-
- The Royal College of Psychiatrists will retain the copyright on the scale.
-
- You will retain all rights to any data generated by use of the scale.
- You will pay Cambridge University Press $0 for this permission.
-
- The scale may not be amended and must be reproduced in full and the following acknowledgement must be included in all versions:

©2008, 2011 The Royal College of Psychiatrists. Williams JBW, Kobak KA. Development and reliability of a structured interview guide for the Montgomery-Asberg Depression Rating Scale (SIGMA). Br J Psychiatry 2008; 192: 52-58. Brianne Brown, PsyD, contributed to this revision. Written permission must be obtained from Cambridge University Press for copying, distribution to others, for replication (in print, online or by any other medium), and translations.

Many thanks and best wishes.

Yours sincerely,

Adam Hirschberg
Senior Business Development Representative
Cambridge University Press

# Efficacy and Safety of Single Subanesthetic S-ketamine in Boosting Efficacy of Oral Antidepressants for Major Depressive Disorder: A Randomized Double-Blind Midazolam-Controlled Pilot Trial

Statistical Analysis Plan

April 1, 2021, Version 1.0

Principle investigator: Gang Wang

Sponsor: Beijing Key Laboratory of Mental Disorders, National Clinical Research Center for Mental Disorders & National Center for Mental Disorders, Beijing Anding Hospital, Capital Medical University, Beijing, China; Advanced Innovation Center for Human Brain Protection, Capital Medical University, Beijing, China

Drafted by: Jia Zhou, Beijing Key Laboratory of Mental Disorders, National Clinical Research Center for Mental Disorders & National Center for Mental Disorders, Beijing Anding Hospital, Capital Medical University, Beijing, China; Advanced Innovation Center for Human Brain Protection, Capital Medical University, Beijing, China

Edited and Verified by Haibo Wang, Peking University Clinical Research Institute, Peking University First Hospital, Beijing, China

# Table of Contents

1. Abbreviations ..... 1
2. Introduction ..... 2
3. Study overview ..... 2
3.1 Objectives ..... 2
3.2 Trial design ..... 2
3.3 Study participants ..... 2
3.4 Sample size ..... 3
3.5 Randomization and blinding ..... 3
3.6 Interventions ..... 4
4. Study outcomes ..... 4
4.1 Primary outcomes ..... 4
4.2 Secondary outcomes ..... 4
4.3 Safety outcomes ..... 4
5. Analysis data set ..... 5
6. Missing data analysis ..... 5
7. Statistical analysis ..... 5
7.1 General principles of statistical analysis ..... 5
7.2 Study completion summary and population dataset ..... 6
7.3 Baseline demographic and clinical characteristics ..... 6
7.4 Outcome analysis ..... 6
7.5 Safety analysis ..... 7
8. Statistical analysis results (table design) ..... 7
8.1 Study completion summary and population dataset ..... 7
8.2 Demographic and clinical characteristics at baseline ..... 8
8.3 Outcome analyses ..... 11
8.4 Safety analysis ..... 14

# 1. Abbreviations

| | |
|---|---|
| AE | Adverse events |
| ALT | Serum alanine aminotransferase |
| AST | Serum glutamic-oxalacetic transaminase |
| BMI | Body Mass Index |
| BPRS-4 | Brief Psychiatric Rating Scale |
| CADSS | Clinician-Administered Dissociative States Scale |
| $\chi^2$ | Chi-square |
| CI | Confidence interval |
| CGI | Clinical Global Impression |
| FAD | **F**luctuating symptoms (re-emergence of initial symptoms/episode or onset of a new episode) occurrs during adequate **A**DT in patients with M**D**D |
| DSM-5 | Diagnostic and Statistical Manual of Mental Disorders, Fifth Edition |
| FAS | Full Analysis Set |
| FIBSER | Frequency, Intensity, and Burden of Side Effects Rating |
| FT3 | Triiodothyronine |
| FT4 | Thyroxine |
| GGT | Gamma-glutamyl transferase |
| HAMD-17 | 17-item Hamilton Rating Scale for Depression |
| IRB | Institutional Review Board |
| ITT | intention-to-treat |
| LOCF | last observation carried forward |
| MADRS | Montgomery-Åsberg Depression Rating Scale |
| MAX | Maximum |
| MDD | Major depressive disorder |
| MIN | Maximum |
| N | Number |
| PPS | Per-protocol Set |
| Q1 | Quartile 1 |
| Q3 | Quartile 3 |
| RCT | Randomized controlled trial |
| SAS | Statistical analysis system |
| SD | Standard Deviation |
| SS | Safety Analysis Set |
| TSH | Thyroid stimulating hormone |
| UKU | Udvalg for Kliniske Undersogelser |
| ULN | Upper limit of normal |
| VAS | Visual Analogue Score |
| YMRS | Young Mania Rating Scale |

# 2. Introduction

This is a statistical analysis plan for "Efficacy and Safety of Single Subanesthetic S-ketamine in Boosting Efficacy of Oral Antidepressants for Major Depressive Disorder: A Randomized Double-Blind Midazolam-Controlled Pilot Trial (hereafter referred to as "the study")." This plan was prepared based on the study protocol (hereafter referred to as "the protocol") of the study.

Institutional Review Board (IRB) of Beijing Anding Hospital approved the study protocol. The study was conducted in accordance with the ethical principles of the Declaration of Helsinki, Good Clinical Practices, and applicable regulatory requirements. All patients provided written informed consent before participating in the study. The study protocol was registered on Chinese Clinical Trial Registry (ChiCTR2100050335).

# 3. Study overview

## 3.1 Objectives

To assess the efficacy and safety of a single subanesthetic dose of S-ketamine in boosting efficacy of oral antidepressants for treating **F**luctuating symptoms occurring during adequate **A**DT in patients with M**D**D (FAD).

## 3.2 Trial design

The study was a single-center, double-blind, randomized controlled pilot trial with balanced randomization (1:1) conducted at Beijing Anding Hospital, Capital Medical University. All patients with FAD were randomly assigned to receive either a single dose of subanesthetic S-ketamine or midazolam. All participants assigned to S-ketamine-treated group (intervention group) received an intravenous injection of 0.2mg/kg S-ketamine within 40 minutes. The participants assigned to midazolam-controlled group (control group) received an intravenous injection of midazolam (0.045mg/kg) within 40 minutes. Patients were assessed at baseline, 40 mins, 2, 4 and 24 hours, 1, 2, 4 and 6 weeks after injection. The study was initiated on April 2021 and, the recruitment of participants was completed on January 2022.

## 3.3 Study participants

To be eligible, participants should meet all of the following criteria: (1) diagnosed MDD according to Diagnostic and Statistical Manual of Mental Disorders, Fifth Edition (DSM-5) (confirmed by Mini International Neuropsychiatric Interview, Mandarin for China Translation Version 7.0.2); (2) with fluctuating symptoms (VAS-2 score $\leq 5$) after symptom relief and stabilization (VAS-1 score

≥ 8); (3) the most recent episode was stabilized by a single antidepressant treatment, without any adjustments in the kind and dosage of the antidepressant, and the duration of drug interruption during this period was less than 7 days; (4) 17-item Hamilton Rating Scale for Depression (HAMD-17) score ≥ 14. (5) able to understand and comply with study protocol; (6) willing to participate and provide written informed consent.

Exclusion criteria included: 1) presence of a history of treatment-resistant depression (TRD), schizophrenia, bipolar disorder and obsessive-compulsive disorder; 2) persons with a psychotic disorder associated with other diseases; 3) currently with psychotic features; 4) history of severe drug allergy or hypersensitivity reaction; 5) women who are pregnant or breastfeeding; 6) “Yes” to item 4 or 5 of the Columbia-Suicide Severity Rating Scale (C-SSRS) "Suicidal Ideation" during the recent episode; or had suicidal behavior during the recent depressive episode according to the C-SSRS "Suicidal Behavior"; or was at serious risk for suicide according to the investigator's judgment; 7) presence of a history of respiratory disease, such as chronic obstructive pulmonary disease, chronic bronchitis, bronchiectasis, etc.; 8) presence of a history of cardiovascular disease, such as hypertension, various types of angina pectoris, myocardial infarction, etc.; 9) alanine aminotransferase (ALT) or aspartate aminotransferase (AST) or gamma-glutamyl transferase (GGT) above the Upper Limit of Normal (ULN), or total bilirubin above the ULN, where the above abnormalities are clinically significant and assessed by the investigator to be dangerous participating; 10) abnormal testing results with clinical significance for free triiodothyronine (FT3), free thyroxine (FT4) or thyroid stimulating hormone (TSH); 11) contraindications to the use of S-ketamine in the presence of severe risk of elevated blood pressure or intracranial pressure, resting systolic/diastolic blood pressure above 180/100 mmHg, pre-eclampsia and eclampsia, hyperthyroidism, ischemic heart disease; 12) presence of contraindications to the use of midazolam such as hypersensitivity to midazolam or any excipient of midazolam, acute angle-closure glaucoma, and untreated open-angle glaucoma; 13) treatment history of electroconvulsive therapy within the past 3 months; 14) presence of a history of ketamine or S-ketamine or its isomers exposure; 15) presence of a history of substance abuse, drug (e.g., methamphetamine, etc.) use; 16) abnormal and clinically significant findings during the screening period; 17) those who screen positive for novel coronavirus nucleic acid; 18) there are other conditions that, in the opinion of the investigator, are inappropriate for participation in this trial.

## 3.4 Sample size

Since this study was a pilot study, as we knew there was no referring literature for estimating effect size. Therefore, no formal sample size calculation was performed for this pilot study. We planned to recruit a total of 30 MDD patients (15 participants in each group). The effect-size data from this pilot study would be used to estimate the sample size of a full randomized controlled trial (RCT) in the future.

## 3.5 Randomization and blinding

Eligible patients were randomly assigned in a 1:1 ratio to receive either single S-ketamine i.v. or single midazolam i.v. for the same duration of 40 minutes. Randomization was performed using a

computer-generated block randomization scheme (block size, 4) using SAS 9.4 (SAS Institute Inc., Cary, NC, USA) by a statistician who is not involved in the study. The generated randomization sequence was concealed in an opaque envelope. The independent assistant who is not involved in the study will be responsible for packing and labeling the prescribed medication. Study investigators, staff, participants, and all outcome assessors were blinded with respect to treatment assignment.

### 3.6 Interventions

The infusion process was performed at Beijing Anding Hospital, Capital Medical University. All subjects were admitted to research ward specific to clinical trials one day before the infusion day and started 6-hour preoperative fasting and water deprivation. On infusion day, 0.2 mg/kg S-ketamine and 0.045 mg/kg midazolam was intravenously given to S-ketamine-treated group and midazolam-controlled group, separately. Both medications were dissolved in 50 ml normal saline and administered for 40 minutes using a syringe pump. Safety checks were performed before and after injection in accordance with the safety check procedure to ensure the safety of the participants. Subjects were permitted to discharge after completing the 24-hour follow-up visit after infusion. During hospitalization, all subjects should comply with relevant management system, take standard meals (uniform light diet), and should not enter and leave the ward at will.

## 4. Study outcomes

### 4.1 Primary outcomes

The primary outcome was rate of response at 2 weeks, namely the proportion of responders who had ≥50% decrease in their scores on the Montgomery-Åsberg Depression Rating Scale (MADRS) at 2 weeks.

### 4.2 Secondary outcomes

Rate of response at 6 weeks, namely the proportion of responders who had ≥50% decrease in their scores on MADRS from baseline at 6 weeks

Rates of remission at 2 and 6 weeks, namely the proportion of individuals who met remission criteria (MADRS total score ≤10) at 2 and 6 weeks;

Change in MADRS score from baseline at each visit from baseline;

Change in Clinical Global Impression (CGI) score from baseline at each visit.

### 4.3 Safety outcomes

Safety evaluation included adverse events (AE), vital signs, the Clinician-Administered Dissociative States Scale (CADSS), the 4-item positive symptom subscale of the Brief Psychiatric

Rating Scale (BPRS-4), the Udvalg for Kliniske Undersogelser (UKU) , Frequency, Intensity, and Burden of Side Effects Rating (FIBSER), and the Young Mania Rating Scale (YMRS). AEs related to the study were judged by a medically qualified investigator and followed until resolution (or if the event was regarded stable).

# 5. Analysis data set

Full Analysis Set (FAS): According to the principle of intention to treat (ITT), the full analysis set is defined as a population of all randomized subjects excluding 1) subjects who have not started study treatment, and 2) subjects without any post-treatment efficacy evaluation.

Per Protocol Set (PPS): The per protocol set, as a subset of the ITT population, are defined as the participants who have no major protocol violations (the eligibility/exclusion criterion, intervention/control treatment, and the completion of primary outcome follow-up)..

Safety Analysis Set (Safety Set, SS): The safety analysis set is defined as a population of all randomized subjects excluding 1) subjects who have never received treatment and 2) subjects without any safety data.The analysis of primary and secondary outcomes are performed based on the FAS and the PPS dataset, and the conclusions will be made mainly based on the FAS dataset. All baseline data analyses are performed on the basis of the FAS and safety assessments are performed on the SS dataset.

# 6. Missing data analysis

For participants who were lost to follow-up at 6 weeks, MADRS score was imputed by the last observation carried forward (LOCF) method in FAS analysis. For other missing data, no missing value imputation was performed.

# 7. Statistical analysis

## 7.1 General principles of statistical analysis

In consultation with the principal investigator, a professional statistician drafted and developed the final version of statistical analysis plan (SAP) prior to database locking.

All the analyses will be performed with SAS statistical software, version 9.4 (SAS Institute, license number: 11202165).

All tests of statistical significance are two-sided, and a *P* value of $<0.05$ will be considered statistically significant (unless otherwise specified).

All analyses will be done according to the intention-to-treat (ITT) principle. The analysis of primary

and secondary outcomes are performed based on the FAS and the PPS dataset, and the conclusions will be made mainly based on the FAS dataset. SS dataset analysis is used for safety evaluation. For participants who switched to other group after randomization, they will be kept in the groups to which they were originally allocated in FAS dataset according to ITT principle.

Continuous variables will be presented with mean, standard deviation (normally distributed), or median (interquartile range). Categorical variables will be presented with the number and percentages. Baseline demographical and clinical characteristics will be shown and compared between the intervention and control groups. The differences of continuous variables will be compared using Student's *t* test for normally distributed variables or the Mann-Whitney U test for non-normally distributed variables. Comparisons on qualitative variables will be done using the $\chi^2$ test or Fisher's exact test, as appropriate.

## 7.2 Study completion summary and population dataset

The number of participants in FAS/PPS/SS dataset will be shown by intervention and control group. The participants who were not included in the FAS, PPS or SS dataset and the reasons for exclusion will be listed. The number of participants enrolled, completed the trial and withdrawn from the trial will be summarized.

## 7.3 Baseline demographic and clinical characteristics

Baseline characteristics will be compared between intervention and control group to evaluate the comparability, including demographic characteristics (age, gender, ethnicity, education, marriage, BMI), behavior characteristics (smoking, alcohol drinking, drug abuse), MDD medical history (history of psychiatric disorders, number of MDD episodes, total course of MDD) and current MDD episodes (first-episode or not, duration of recent episode, duration of ADT for recent episode, duration of ongoing ADT with current antidepressant, duration of mataining stable after symptom relief with current antidepressant [VAS-1 score ≥ 8] , duration of fluctuating symptoms with current antidepressant [VAS-2 score ≤ 5], VAS-1 score, VAS-2 score, HAMD-17 score, MADRS score, the kind and dosage of antidepressant).

## 7.4 Outcome analysis

Outcome analyses will be done in both FAS and PPS dataset.
(1) Primary outcome: rate of response (defined as ≥50% reduction from baseline MADRS score) at 2 weeks will be compared using the $\chi 2$ test or Fisher's exact test as required.
(2) Secondary outcomes 1: rate of response at 6 weeks, rates of remission at 2 and 6 weeks (defined as a MADRS score ≤ 10) will be compared using the $\chi 2$ test or Fisher's exact test as required.
(3) Secondary outcomes 2: change in MADRS score over time will be analyzed using two-way repeated-measures ANOVA for group and time interaction effects.
(4) Secondary outcomes 3: change in CGI score over time will be analyzed using two-way repeated-measures ANOVA for group and time interaction effects.

### 7.5 Safety analysis

The safety evaluation will be analyzed on the SS dataset.
The number of adverse events of the two groups, as well as the incidence of AE/SAE will be shown in tables.
Chi-square test or the Fisher exact test will be used to compare incidence of AE/SAE and the proportion of subjects with “new” or “worsened” symptoms on the UKU between the two groups. The difference in dissociative symptoms assessed with the CADSS, BPRS-4, YMRS, FIBSER, will be compared between two groups using wilcoxon rank sum test at different visiting timepoints.

## 8. Statistical analysis results (table design)

### 8.1 Study completion summary and population dataset

Table 1 Population dataset by intervention and control group1

| FAS | | | PPS | | | SS (Actual Group) | | |
|---|---|---|---|---|---|---|---|---|
| intervention group | control group | Total | intervention group | control group | Total | intervention group | control group | Total |
| | | | | | | | | |

Table 2 Listing of participants not included in the FAS/PPS/SS dataset2

| ID | Enrolment date | Reason of exclusion | group | Included in analysis dataset | | |
|---|---|---|---|---|---|---|
| | | | | PPS | FAS | SS |
| | | | | | | |

Table 3 The summary of study completion

| | Items | intervention group | control group | Total |
|---|---|---|---|---|
| Screening population | | | | |
| | Enrolment and randomization | | | |
| Excluded from FAS | | | | |
| | Failure to meet inclusion/exclusion criteria | | | |
| | Withdrawn after randomization | | | |
| Withdrawn during the study | | | | |
| | Upon requests from participants or their representatives | | | |

| | |
|---|---|
| | Lost to follow-up |
| | Crossover |
| | Protocol deviation |
| | Other |
| Safety analysis set | |
| | SS (Actual Group) |
| Outcome analysis set | |
| | FAS |
| | PPS |

## 8.2 Demographic and clinical characteristics at baseline

Table 4 Demographic characteristics at baseline3

| Items | Indicators | Total | Intervention group | Control group | *P* |
|---|---|---|---|---|---|
| Age (years) | N (Missing) | | | | |
| | Mean (SD) | | | | |
| | Median | | | | |
| | Q1, Q3 | | | | |
| | Min, Max | | | | |
| Body mass index (BMI, $Kg/m^2$) | N (Missing) | | | | |
| | Mean (SD) | | | | |
| | Median | | | | |
| | Q1, Q3 | | | | |
| | Min, Max | | | | |
| Gender | Male, n (%) | | | | |
| | Female, n (%) | | | | |
| Ethnicity | Han, n (%) | | | | |
| | Other, n (%) | | | | |
| Education level (Years) | ≤12, n (%) | | | | |
| | >12, n (%) | | | | |
| Marriage | Unmarried | | | | |
| | Married | | | | |

Table 5 Behavioural characteristics and family history at baseline

| Items | Indicators | Total | Intervention group | Control group | *P* |
|---|---|---|---|---|---|
| Alcohol history | Yes, n (%) | | | | |
| | No, n (%) | | | | |
| Smoking history | Yes, n (%) | | | | |
| | No, n (%) | | | | |
| Drug abuse | Yes, n (%) | | | | |
| | No, n (%) | | | | |

| Items | Indicators | Total | Intervention group | Control group | *P* |
|---|---|---|---|---|---|
| Family history of psychiatric illness | Yes, n (%) | | | | |
| | No, n (%) | | | | |

4

Table 7 MDD history and current MDD episodes at baseline

| Items | Indicators | Total | Intervention group | Control group | *P* |
|---|---|---|---|---|---|
| Age at onset of MDD (years) | N (Missing) | | | | |
| | Mean (SD) | | | | |
| | Median | | | | |
| | Q1, Q3 | | | | |
| | Min, Max | | | | |
| Total course of MDD (years) | N (Missing) | | | | |
| | Mean (SD) | | | | |
| | Median | | | | |
| | Q1, Q3 | | | | |
| | Min, Max | | | | |
| Number of MDD episodes | N (Missing) | | | | |
| | Mean (SD) | | | | |
| | Median | | | | |
| | Q1, Q3 | | | | |
| | Min, Max | | | | |
| Prior psychiatric hospitalization (times) | N (Missing) | | | | |
| | Mean (SD) | | | | |
| | Median | | | | |
| | Q1, Q3 | | | | |
| | Min, Max | | | | |
| First-episode | Yes, n (%) | | | | |
| | No, n (%) | | | | |
| Screening HAMD-17 score | N (Missing) | | | | |
| | Mean (SD) | | | | |
| | Median | | | | |
| | Q1, Q3 | | | | |
| | Min, Max | | | | |
| Screening MADRS score | N (Missing) | | | | |
| | Mean (SD) | | | | |
| | Median | | | | |
| | Q1, Q3 | | | | |
| | Min, Max | | | | |
| Duration of recent episode (weeks) | N (Missing) | | | | |

| Items | Indicators | Total | Intervention group | Control group | *P* |
|---|---|---|---|---|---|
| | Mean (SD) | | | | |
| | Median | | | | |
| | Q1, Q3 | | | | |
| | Min, Max | | | | |
| Duration of ADT for recent episode (weeks) | N (Missing) | | | | |
| | Mean (SD) | | | | |
| | Median | | | | |
| | Q1, Q3 | | | | |
| | Min, Max | | | | |
| Duration of ongoing ADT with current antidepressant (weeks) | N (Missing) | | | | |
| | Mean (SD) | | | | |
| | Median | | | | |
| | Q1, Q3 | | | | |
| | Min, Max | | | | |
| Duration of mataining stable after symptom relief with current antidepressant (weeks) | N (Missing) | | | | |
| | Mean (SD) | | | | |
| | Median | | | | |
| | Q1, Q3 | | | | |
| | Min, Max | | | | |
| Duration of fluctuating symptoms with current antidepressant (weeks) | N (Missing) | | | | |
| | Mean (SD) | | | | |
| | Median | | | | |
| | Q1, Q3 | | | | |
| | Min, Max | | | | |
| Screening VAS-1 score | N (Missing) | | | | |
| | Mean (SD) | | | | |
| | Median | | | | |
| | Q1, Q3 | | | | |
| | Min, Max | | | | |
| Screening VAS-2 score | N (Missing) | | | | |
| | Mean (SD) | | | | |
| | Median | | | | |
| | Q1, Q3 | | | | |
| | Min, Max | | | | |
| Antidepressant | Duloxetine, n (%) | | | | |
| | Escitalopram, n (%) | | | | |

| Items | Indicators | Total | Intervention group | Control group | *P* |
|---|---|---|---|---|---|
| | Sertraline, n (%) | | | | |
| | Fluvoxamine, n (%) | | | | |
| | Fluoxetine, n (%) | | | | |
| | Venlafaxine, n (%) | | | | |
| | Mirtazapine, n (%) | | | | |
| | Other, n (%) | | | | |
| Class of antidepressant | Serotonin-norepinephrine reuptake inhibitor, n (%) | | | | |
| | Selective serotonin reuptake inhibitor, n (%) | | | | |
| | Atypical antidepressants, n (%) | | | | |

## 8.3 Outcome analyses

1. Primary outcome: Rate of response at 2 weeks

Table 9 Rate of response at 2 weeks by intervention and control group5

| | | FAS | | PPS | |
|---|---|---|---|---|---|
| Items | Indicators | Intervention group | Control group | Intervention group | Control group |
| Rate of response at 2 weeks | No, n (%) | | | | |
| | Yes, n (%) | | | | |
| | N (Missing) | | | | |
| | Difference and 95%CI | | | | |
| | Test statistics | | | | |
| | P values | | | | |

2. Secondary outcomes: Rate of response at 6 weeks

Table 11 Rate of response at 6 weeks by intervention and control group6

| | | FAS | | PPS | |
|---|---|---|---|---|---|
| Item | Indicators | Intervention group | Control group | Intervention group | Control group |
| Rate of response at 6 weeks | No, n (%) | | | | |
| | Yes, n (%) | | | | |
| | N (Missing) | | | | |
| | Difference and 95%CI | | | | |
| | Test statistics | | | | |
| | P values | | | | |

3. Secondary outcomes: Rates of remission at 2 and 6 weeks

Table 10 Rates of remission at 2 and 6 weeks by intervention and control group7

| | | FAS | | PPS | |
|---|---|---|---|---|---|
| Items | Indicators | Intervention group | Control group | Intervention group | Control group |
| Rates of remission at 2 weeks | No, n (%) | | | | |
| | Yes, n (%) | | | | |
| | N (Missing) | | | | |
| | Difference and 95%CI | | | | |
| | Test statistics | | | | |
| | P values | | | | |
| Rates of remission at 6 weeks | No, n (%) | | | | |
| | Yes, n (%) | | | | |
| | N (Missing) | | | | |
| | Difference and 95%CI | | | | |
| | Test statistics | | | | |
| | P values | | | | |

4. Secondary outcomes: Change in MADRS score over time

Table 12 Change in MADRS score over time by intervention and control group8

| | | FAS | | PPS | |
|---|---|---|---|---|---|
| Visiting timepoints | Indicators | Intervention group | Control group | Intervention group | Control group |
| Baseline | N (Missing) | | | | |
| | Mean (SD) | | | | |
| | Median | | | | |
| | Q1, Q3 | | | | |
| | Min, Max | | | | |
| | Difference and 95%CI | | | | |
| | Test statistics | | | | |
| | P values | | | | |
| 2 hours | N (Missing) | | | | |
| | Mean (SD) | | | | |
| | Median | | | | |
| | Q1, Q3 | | | | |
| | Min, Max | | | | |
| | Difference and 95%CI | | | | |
| | Test statistics | | | | |
| | P values | | | | |
| …….. | ……….. | | | | |

5. Secondary outcomes: Change in CGI score over time

Table 13 Change in CGI score over time by intervention and control group

| | | FAS | | PPS | |
|---|---|---|---|---|---|
| Visiting timepoints | Indicators | Intervention group | Control group | Intervention group | Control group |
| Baseline | N (Missing) | | | | |
| | Mean (SD) | | | | |
| | Median | | | | |
| | Q1, Q3 | | | | |
| | Min, Max | | | | |
| | Difference and 95%CI | | | | |
| | Test statistics | | | | |
| | P values | | | | |
| 2 hours | N (Missing) | | | | |
| | Mean (SD) | | | | |
| | Median | | | | |
| | Q1, Q3 | | | | |
| | Min, Max | | | | |
| | Difference and 95%CI | | | | |
| | Test statistics | | | | |
| | P values | | | | |

| | | FAS | | PPS | |
|---|---|---|---|---|---|
| Visiting timepoints | Indicators | Intervention group | Control group | Intervention group | Control group |
| …….. | ……….. | | | | |

## 8.4 Safety analysis

1. Udvalg for Kliniske Undersogelser (UKU)

Table 15 The numbers of patients with "new" or "worsened" symptoms on the UKU between the two groups.

| | | FAS | | PPS | |
|---|---|---|---|---|---|
| Items | Indicators | Intervention group | Control group | Intervention group | Control group |
| Adverse events | No, n (%) | | | | |
| | Yes, n (%) | | | | |
| | N (Missing) | | | | |
| | Difference and 95%CI | | | | |
| | Test statistics | | | | |
| | P values | | | | |

2. Clinician-Administered Dissociative States Scale (CADSS)

Table 16 Change in CADSS score over time by intervention and control group

| | | FAS | | PPS | |
|---|---|---|---|---|---|
| Visiting timepoints | Indicators | Intervention group | Control group | Intervention group | Control group |
| Baseline | N (Missing) | | | | |
| | Mean (SD) | | | | |
| | Median | | | | |
| | Q1, Q3 | | | | |
| | Min, Max | | | | |
| | Difference and 95%CI | | | | |
| | Test statistics | | | | |
| | P values | | | | |
| 2 hours | N (Missing) | | | | |
| | Mean (SD) | | | | |
| | Median | | | | |
| | Q1, Q3 | | | | |
| | Min, Max | | | | |
| | Difference and 95%CI | | | | |
| | Test statistics | | | | |
| | P values | | | | |

| | | FAS | | PPS | |
|---|---|---|---|---|---|
| Visiting timepoints | Indicators | Intervention group | Control group | Intervention group | Control group |
| …….. | ……….. | | | | |

3. the Brief Psychiatric Rating Scale (BPRS-4)

Table 17 Change in BPRS-4 score over time by intervention and control group

| | | FAS | | PPS | |
|---|---|---|---|---|---|
| Visiting timepoints | Indicators | Intervention group | Control group | Intervention group | Control group |
| Baseline | N (Missing) | | | | |
| | Mean (SD) | | | | |
| | Median | | | | |
| | Q1, Q3 | | | | |
| | Min, Max | | | | |
| | Difference and 95%CI | | | | |
| | Test statistics | | | | |
| | P values | | | | |
| 2 hours | N (Missing) | | | | |
| | Mean (SD) | | | | |
| | Median | | | | |
| | Q1, Q3 | | | | |
| | Min, Max | | | | |
| | Difference and 95%CI | | | | |
| | Test statistics | | | | |
| | P values | | | | |
| …….. | ……….. | | | | |

4. Frequency, Intensity, and Burden of Side Effects Rating (FIBSER)

Table 18 Change in FIBSER score over time by intervention and control group

| | | FAS | | PPS | |
|---|---|---|---|---|---|
| Visiting timepoints | Indicators | Intervention group | Control group | Intervention group | Control group |
| Baseline | N (Missing) | | | | |
| | Mean (SD) | | | | |
| | Median | | | | |
| | Q1, Q3 | | | | |
| | Min, Max | | | | |
| | Difference and 95%CI | | | | |
| | Test statistics | | | | |
| | P values | | | | |
| 2 hours | N (Missing) | | | | |

| | | FAS | | PPS | |
|---|---|---|---|---|---|
| Visiting timepoints | Indicators | Intervention group | Control group | Intervention group | Control group |
| | Mean (SD) | | | | |
| | Median | | | | |
| | Q1, Q3 | | | | |
| | Min, Max | | | | |
| | Difference and 95%CI | | | | |
| | Test statistics | | | | |
| | P values | | | | |
| …….. | ……….. | | | | |

5. Young Mania Rating Scale (YMRS)

Table 18 Change in YMRS score over time by intervention and control group

| | | FAS | | PPS | |
|---|---|---|---|---|---|
| Visiting timepoints | Indicators | Intervention group | Control group | Intervention group | Control group |
| Baseline | N (Missing) | | | | |
| | Mean (SD) | | | | |
| | Median | | | | |
| | Q1, Q3 | | | | |
| | Min, Max | | | | |
| | Difference and 95%CI | | | | |
| | Test statistics | | | | |
| | P values | | | | |
| 2 hours | N (Missing) | | | | |
| | Mean (SD) | | | | |
| | Median | | | | |
| | Q1, Q3 | | | | |
| | Min, Max | | | | |
| | Difference and 95%CI | | | | |
| | Test statistics | | | | |
| | P values | | | | |
| …….. | ……….. | | | | |
