# Supplementary material for: Esketamine vs Midazolam in Boosting the Efficacy of Oral Antidepressants for Major Depressive Disorder: A Pilot Randomized Clinical Trial
Source: JAMA Netw Open. 2023 Aug 14;6(8):e2328817. doi: 10.1001/jamanetworkopen.2023.28817 (PMC10425830; doi:10.1001/jamanetworkopen.2023.28817)
Supplement: Supplement 2. — eTable 1. Rates of Remission at Different Visits by Intervention and Control Group eFigure. The Least-Square Mean Reduction of MADRS Scores From Baseline to 6 Weeks at Each Visit by Intervention and Control Group eTable 2. Reduction of CGI-S Score From Baseline at Different Visits by Intervention and Control Group [file jamanetwopen-e2328817-s002.pdf]

Supplemental Online Content

Xiao C, Zhou J, Li A, et al. Esketamine vs midazolam in boosting the efficacy of oral antidepressants for major depressive disorder: a pilot randomized clinical trial. *JAMA Netw Open*. 2023;6(8):e2328817. doi:10.1001/jamanetworkopen.2023.28817

eTable 1. Rates of Remission at Different Visits by Intervention and Control Group
eFigure. The Least-Square Mean Reduction of MADRS Scores From Baseline to 6 Weeks at Each Visit by Intervention and Control Group
eTable 2. Reduction of CGI-S Score From Baseline at Different Visits by Intervention and Control Group

This supplemental material has been provided by the authors to give readers additional information about their work.

© 2023 Xiao C et al. *JAMA Network Open.*

eTable 1. Rates of remission at different visits by intervention and control group, n (%)

Visiting timepoints	Midazolam-controlled group	S-ketamine-treated group	*P*
2 hours	3 (20.0)	8 (53.3)	0.06
4 hours	3 (20.0)	10 (66.7)	0.01
24 hours	4 (26.7)	9 (60.0)	0.07
1 week	3 (20.0)	7 (46.7)	0.12
2 weeks	2 (13.3)	6 (40.0)	0.10
4 weeks	1 (6.7)	8 (53.3)	0.005
6 weeks	4 (26.7)	9 (60.0)	0.07

© 2023 Xiao C et al. *JAMA Network Open.*

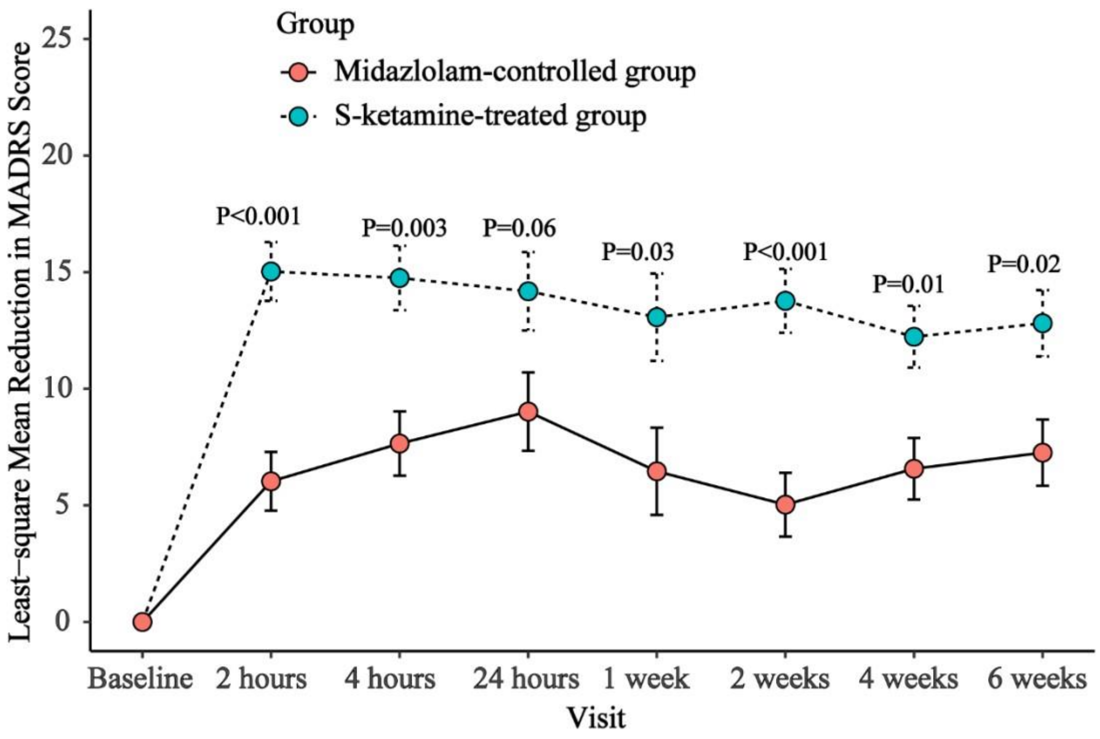

eFigure. The least-square mean reduction of MADRS scores from baseline to six weeks at each visit by intervention and control group.

Note: Error bars indicated standard error. This analysis was performed using a covariance (ANCOVA) model with baseline MADRS score (the Montgomery-Åsberg Depression Rating Scale; range: 0-60; higher scores indicating more severe depressive symptom) and the number of MDD episodes as covariates. There was one patient in the S-ketamine-treated group dropped out at the end of 1 week. He received 0.2 mg/kg S-ketamine infusion, and left the city due to job changes. Missing data of MADRS score was imputed using the last observation carried forward (LOCF).

© 2023 Xiao C et al. *JAMA Network Open.*

eTable 2. Reduction of CGI-S score from baseline at different visits by intervention and control group, Median (IQR)

Visiting timepoints	Midazolam-controlled group	S-ketamine-treated group	P
2 hours	1.0 (0.0-1.0)	3.0 (2.0-3.0)	<.001
4 hours	1.0 (0.0-2.0)	3.0 (3.0-4.0)	<.001
24 hours	1.0 (0.0-2.0)	3.0 (2.0-4.0)	<.001
1 week	0.0 (0.0-2.0)	3.0 (2.0-4.0)	0.001
2 weeks	0.0 (0.0-1.0)	3.0 (2.0-4.0)	<.001
4 weeks	1.0 (0.0-1.0)	3.0 (2.0-4.0)	<.001
6 weeks	1.0 (0.0-1.0)	3.0 (2.0-4.0)	0.001

© 2023 Xiao C et al. *JAMA Network Open.*
