# Supplementary material for: Esketamine vs Midazolam in Boosting the Efficacy of Oral Antidepressants for Major Depressive Disorder: A Pilot Randomized Clinical Trial
Source: JAMA Netw Open. 2023 Aug 14;6(8):e2328817. doi: 10.1001/jamanetworkopen.2023.28817 (PMC10425830; doi:10.1001/jamanetworkopen.2023.28817)
Supplement: Supplement 3. — Data Sharing Statement [file jamanetwopen-e2328817-s003.pdf]

# Data Sharing Statement

Xiao. Esketamine vs Midazolam in Boosting the Efficacy of Oral Antidepressants for Major Depressive Disorder. *JAMA Netw Open*. Published August 14, 2023.
doi:10.1001/jamanetworkopen.2023.28817

**Data**
**Data available:** No
